# Barriers to simultaneous multilocus integration in *Bacillus subtilis* tumble down: development of a straightforward screening method for the colorimetric detection of one-step multiple gene insertion using the CRISPR-Cas9 system

**DOI:** 10.1186/s12934-023-02032-2

**Published:** 2023-01-31

**Authors:** Jordi Ferrando, Oriana Filluelo, Daniel R. Zeigler, Pere Picart

**Affiliations:** 1 Microbiology Section, Department of Biology, Healthcare and Environment, Faculty of Pharmacy and Food Sciences, Universitat de Barcelona, Barcelona, Catalonia Spain; 2 Subtilogic LLC, Columbus, OH USA

**Keywords:** *B. subtilis*, CRISPR-Cas9, Colorimetric screening, Genome engineering, Multigene insertion

## Abstract

**Background:**

Despite recent advances in genetic engineering tools for effectively regulating and manipulating genes, efficient simultaneous multigene insertion methods have not been established in *Bacillus subtilis*. To date, multilocus integration systems in *B. subtilis*, which is one of the main industrial enzyme producers and a GRAS (generally regarded as safe) microbial host, rely on iterative rounds of plasmid construction for sequential insertions of genes into the *B. subtilis* chromosome, which is tedious and time consuming.

**Results:**

In this study, we present development and proof-of-concept of a novel CRISPR-Cas9-based genome-editing strategy for the colorimetric detection of one-step multiple gene insertion in *B. subtilis*. First, up to three copies of the *crtMN* operon from *Staphylococcus aureus*, encoding a yellow pigment, were incorporated at three ectopic sites within the *B. subtilis* chromosome, rendering engineered strains able to form yellow colonies. Second, a single CRISPR-Cas9-based plasmid carrying a highly specific single guide RNA (sgRNA) targeting *crtMN* operon and a changeable editing template was constructed to facilitate simultaneous insertion of multiple gene-copies through homology-directed repair (HDR). Upon transformation of engineered strains with engineered plasmids, strains harboring up to three gene copies integrated into the chromosome formed white colonies because of the removal of the *crtMN* operon, clearly distinguishable from yellow colonies harboring undesired genetic modifications. As a result, construction of a plasmid-less, marker-free, high-expression stable producer *B. subtilis* strain can be completed in only seven days, demonstrating the potential that the implementation of this technology may bring for biotechnology purposes.

**Conclusions:**

The novel technology expands the genome-editing toolset for *B. subtilis* and means a substantial improvement over current methodology, offering new application possibilities that we envision should significantly boost the development of *B. subtilis* as a chassis in the field of synthetic biology.

**Supplementary Information:**

The online version contains supplementary material available at 10.1186/s12934-023-02032-2.

## Background

*Bacillus* is a genus of gram-positive, rod-shaped bacteria, which due to their wide distribution, safety in work, ease of cultivation, and susceptibility to genetic transformation, have been widely used to produce heterologous proteins [1, 2]. *B. subtilis* and related *Bacillus* strains are the dominant enzyme-producing microorganisms in applied and industrial microbiology owing to their ability to secrete enzymes at very high levels [3–5], in addition to their extensive use to produce drug precursors, platform compounds, biofuels and biopolymers [6, 7]. To achieve maximum expression of a particular gene in *Bacillus*, it is highly desirable to amplify the copy number of the gene of interest [8]. To this end, a traditional approach involves the introduction of replicative plasmids, where the level of gene expression is dictated by the copy number of the plasmid in the cells [9]. However, the use of antibiotic resistance markers limits their use in industrial applications due to both the genetic instability of many recombinant plasmids in the absence of selection and the restrictions and concerns derived from the massive abuse of antibiotics, promoting the emergence of bacterial resistance.

Protein expression attributed to a single copy of the integrated gene usually results in lower yields of product compared to the use of high copy-number vectors. To circumvent this limitation, the use of a single-crossover integrative vector, which creates direct repeats of the target fragment upon its insertion into the chromosome, offers the possibility of amplifying the integrated plasmid by growing cultures in increasing concentrations of the selective antibiotic and, therefore, amplifying gene dosage in the chromosome [10]. However, although some reports indicate that gene amplification is stable under non-selective conditions [11, 12], other authors show that integrated plasmid copies are gradually lost in the absence of selection [13–15], resulting in unstable strains not suitable for industrial applications. On the other hand, gene-replacement strategies based on double-crossover recombination integrative plasmids allow introduction of mutations that are stable in the absence of ongoing selection. Nevertheless, this approach has the disadvantage that resulting strains have low gene dosage unless multiple rounds of gene insertion are performed [16–18], until reaching expression levels comparable to that of cells carrying multiple copies of a recombinant plasmid. Therefore, the construction of environmentally friendly, marker-free industrial strains of *B. subtilis* with multicopy genes is limited by the availability of selection markers, involving labor-intensive methods of introducing recycling markers. Such methods for optimal marker recycling in *B. subtilis* have been developed based on: (i) counter-selectable markers such as *mazF*, *blaI*, *ysbC* and *uppC* [19–23]; (ii) site-specific recombinase systems such as Cre/LoxP and FLP/FRT [24, 25]; and (iii) the λ-Red phage mediated single-stranded DNA recombination system [26], thus allowing the removal of the selectable marker once the desired chromosome modification is performed in order to reuse it in further rounds of modification. Nevertheless, these methods are still time consuming, laborious, and quite inefficient.

CRISPR-Cas (Clustered Regularly Interspaced Short Palindromic Repeat-CRISPR associated protein) systems, especially with nuclease Cas9, were rapidly adapted for genome editing in *B. subtilis*, thus facilitating the introduction of gene mutations, deletions, and insertions [27–30]. Typically, a single plasmid containing the Cas9 endonuclease is targeted to a specific site by a 20-nucleotide sgRNA also present in the vector. By means of homologous recombination with a plasmid-cloned editing template, this system enables genome editing and cell survival. The introduction of the CRISPR-Cas9 system simultaneously removed the need for selection markers in genome editing and radically increased editing efficiencies, becoming one of the most powerful tools for genome engineering in *B. subtilis* [31–35]. However, to the best of our knowledge, no editing efficiencies have been reported for simultaneous integration of multiple genes in *B. subtilis*, possibly due to the low integration efficiencies achieved during the process.

For the first time, the present study aimed to develop both a CRISPR-Cas9-mediated genomic multigene insertion method in *B. subtilis* and a colorimetric high-throughput screening method for identification of multicopy clones. To this end, the *crtMN* operon encoding yellow pigment from *S. aureus* was first integrated into the *B. subtilis* chromosome at three ectopic sites, thus obtaining a yellowish-pigmented strain. Using a single CRISPR-Cas9-based plasmid harboring a unique high-efficiency target site and changeable editing template, we developed a white/yellow colorimetric screening for white colonies because of the removal of the *crtMN* operon, which if still present leads to the formation of yellow colonies in *B. subtilis*. Thus, an easy, fast, and suitable method to identify white clones for genomic double- (DGI) and triple-gene integration (TGI) in a single step was established. By using this technology, we were able to construct plasmid-less, marker-free stable producer strains harboring up to three copies of an α-amylase gene inserted into the *B. subtilis* genome in only one week. Additionally, we expanded this technology for the fine-tuning of gene expression by switching a constitutive promoter for a xylose-inducible promoter based on the xylose-repressor system. This system extends the repertoire of molecular toolboxes for genetic manipulations and biotechnological endeavors by enabling simultaneous integration of multiple gene copies, which we anticipate will enhance the development of *B. subtilis* as a platform to produce important enzymes and other commodities.

## Results

### Genetic manipulation of colony color in *B. subtilis*

**Fig. 1:**
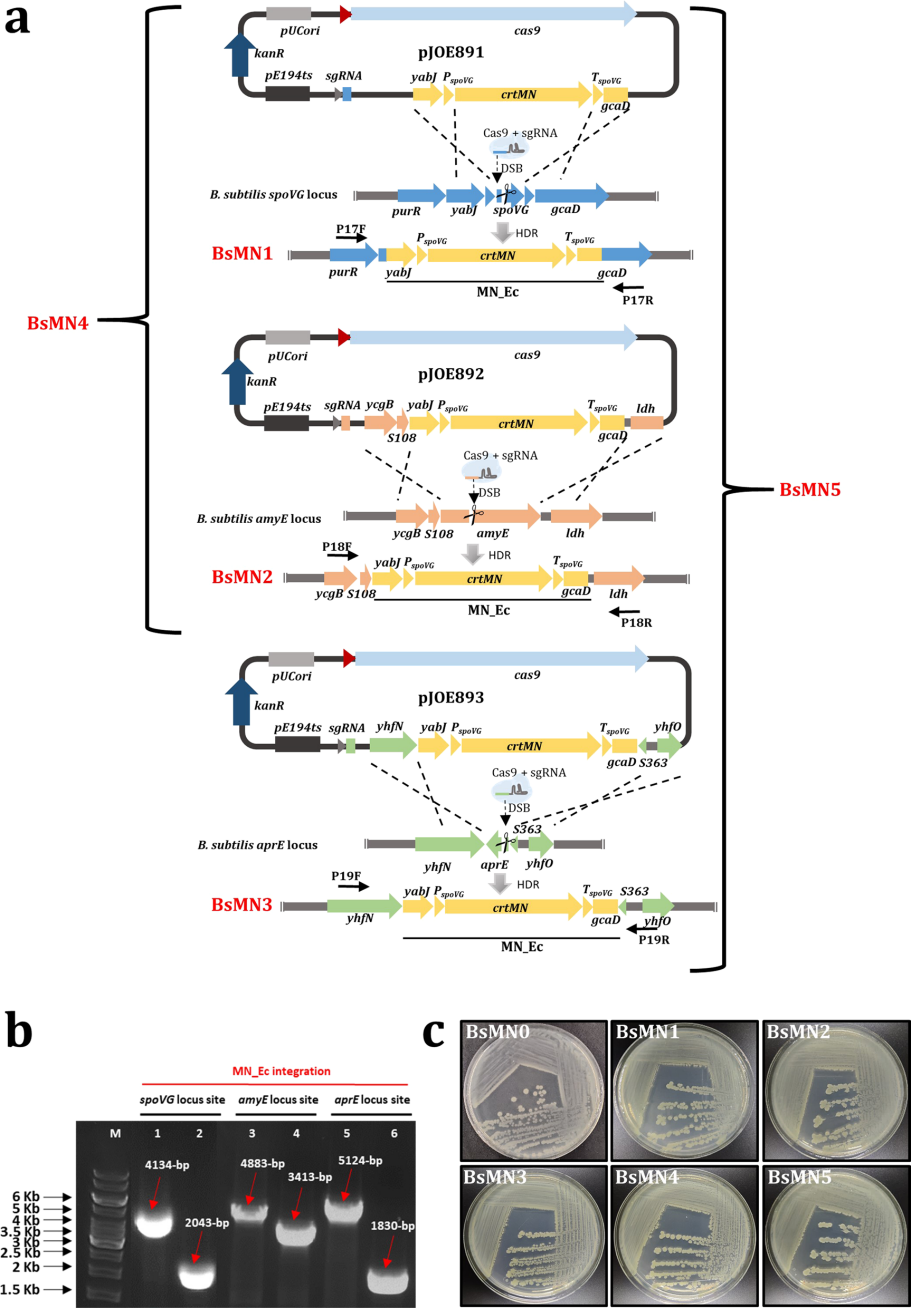
Schematic representation of BsMN1-BsMN5 strains construction system. **a** Integration of MN_Ec at *spoVG* locus using plasmid pJOE891, yielding BsMN1. MN_Ec integration at *amyE* locus using plasmid pJOE892, to yield BsMN2. MN_Ec integration at *aprE* locus using plasmid pJOE893, yielding BsMN3. BsMN4 strain contains two MN_Ec copies integrated at *spoVG* and *amyE* locus sites, whereas BsMN5 contains three MN_Ec copies integrated at *spoVG, amyE* and *aprE* locus sites. DSB: double-strand breaks. **b** Confirmation of the *spoVG*, *amyE* and *aprE* genes replacement for the MN_Ec in BsMN5 strain. Lanes 1, 3 and 5 corresponds to amplification bands of 4134-bp, 4883-bp and 5124-bp using primers P17F/P17R, P18F/P18R and P19F/P19R to verify MN_Ec integrations at *spoVG, amyE* and *aprE* locus sites with gDNA from BsMN5 as template, respectively. Lanes 2, 4 and 6 corresponds to amplifications bands of 2043-bp, 3413-bp and 1830-bp using the same primers with gDNA from BsMN0 as control. M corresponds to the molecular marker weight. **c** LB-agar plates showing white/yellow colonies of strains BsMN0-BsMN5

In *S. aureus*, the yellow C30 carotenoid 4,4′-diaponeurosporene is synthesized by the 2385-bp *crtMN* operon [36]. It has been reported that plasmid-mediated recombinant expression of this operon in *B. subtilis* results in a colony color change from white to yellow [37]. In this study, we wanted to ascertain whether a strain harboring the *crtMN* operon inserted into its chromosome would also produce yellow colonies, as a preliminary step to implement our colorimetric screening method. For this purpose, the pJOE8999 plasmid, which has been widely used for CRISPR-Cas9-mediated engineering in *B. subtilis* [27], was used to exchange the *spoVG* gene of recipient *B. subtilis* strain (BsMN0) with the *crtMN* operon, setting the expression of the encoded yellow C30 carotenoid under the control of the strong *spoVG* promoter (P_*spoVG*_), and flanked by rho-independent transcriptional terminator (T_*spoVG*_) downstream of the same gene [38]. The employed editing vector, denoted as pJOE891, contained a sgRNA targeting the *spoVG* gene and a homology repair expression cassette (MN_Ec) composed of the 530-bp upstream flanking genomic region of *spoVG* followed by the *crtMN* operon and the 535-bp downstream flanking genomic region of *spoVG* (Fig. 1). Upon transformation of BsMN0 with pJOE891, we obtained the BsMN1 strain, which satisfactorily produced the yellow pigment, thus turning the *B. subtilis* colonies from white to yellow in color. Next, a set of 2 vectors was constructed, namely pJOE892 and pJOE893. The first one was engineered to replace the extracellular amylase gene of *B. subtilis* (*amyE*) with MN_Ec, rendering the BsMN2 strain inactive for amylase production. The latter was constructed to facilitate the insertion of the MN_Ec into the already inactivated extracellular protease *aprE* gene to obtain BsMN3 strain. Noticeably, both constructs contained the 530 bp upstream arm and the 535-bp downstream arm flanking genomic region of *spoVG.* Next, iterative genome editing was performed by a successive double- and triple-MN_Ec integration to yield strains BsMN4 and BsMN5, harboring two and three copies of MN_Ec integrated at specific sites, respectively. A comprehensive scheme for the construction of each yellow pigment-producing strain is depicted in Fig. 1a. Using specific primers, the identity of each recombinant strain was demonstrated by PCR verification and Sanger sequencing, showing that MN_Ec was successfully inserted into the *B. subtilis* chromosome (Fig. 1b). Irrespective of the *crtMN* operon copy number, all engineered strains formed yellow-pigmented colonies with a highly similar appearance on LB-agar plates, corroborating the successful expression of the yellow C30-carotenoid (Fig. 1c).

### Establishment of a white/yellow colorimetric screening method

The basis of the CRISPR-Cas9-mediated white/yellow colorimetric screening method proposed in this work is the generation of double-strand breaks (DSBs) at *crtMN* operon target sites in yellow-pigmented strains and their repair through HDR, thus enabling selection for white colonies because of the removal of the *crtMN* operon, which if still present will lead to the formation of yellow colonies. However, it is crucial that DSBs generated by this system are exclusively repaired through HDR because the NHEJ (non-homologous end joining) system may lead to unintended rearrangements at the target region [39], which may consequently interfere with the screening system. Thus, to explore the ability of the unwanted NHEJ system to repair DSBs produced at the *crtMN* operon site, a high-scoring sgRNA sequence targeting the *crtMN* operon (5′-ACCAGAAGATCAAAGAAAAGCGG-3′) was identified using the online sgRNA Designer tool provided by the Broad Institute [40]. This target site was determined to be unique following BLASTN analysis against the *B. subtilis* chromosome, and the closest homolog had 5 mismatches, which thereby prevents off-target effects. Plasmid pJOE8999 was engineered to contain this target sequence yielding pJOE894, which was used to transform BsMN1-BsMN3 strains. The lack of colonies obtained upon transformation (Table 1) demonstrated that the introduction of a DSB at the *crtMN* operon is lethal for the cells unless we provide an editing template, ensuring that NHEJ system will not disturb the colorimetric screening method.

**Table 1:** Summary of efficiency results and CFU for SGI, DGI and TGI using plasmids pJOE894, pJOE895 and pJOE896. Experiments were carried out in triplicates and data are presented as mean values ± standard deviation

Gene integration number	Plasmid	CFU/µg plasmid^a^	No. of Kan^r^ white clones (% white clones)	% Kan^s^ white clones (total no. of colonies tested)^b^	Efficiency for Kan^s^ white clones (%)^c^	Purpose
SGI (*spoVG* locus)	pJOE894^d^	0	0	0	0	NHEJ functionality in *B. subtilis*
	pJOE895^e^	150 ± 21	147 ± 18 (98.2 ± 1.4)	95 (40)	100 (8/8)	*ΔcrtMN* (Q_Ec) knock-in at *spoVG* site
	pJOE896^f^	53 ± 7	49 ± 5 (92.7 ± 2.7)	90 (40)	100 (8/8)	*ΔcrtMN* (Qxyl_Ec) knock-in at *spoVG* site
SGI (*amyE* locus)	pJOE894	0	0	0	0	NHEJ functionality in *B. subtilis*
	pJOE895	147 ± 32	143 ± 30 (97.5 ± 0.6)	92.5 (40)	100 (8/8)	*ΔcrtMN* (Q_Ec) knock-in at *amyE* site
	pJOE896	42 ± 4	38 ± 2 (90 ± 3.8)	87.5 (40)	100 (8/8)	*ΔcrtMN* (Qxyl_Ec) knock-in at *amyE* site
SGI (*aprE* locus)	pJOE894	0	0	0	0	NHEJ functionality in *B. subtilis*
	pJOE895	139 ± 34	137 ± 33 (98.6 ± 0.4)	97.5 (40)	100 (8/8)	*ΔcrtMN* (Q_Ec) knock-in at *aprE* site
	pJOE896	61 ± 11	55 ± 8 (90.6 ± 5.9)	95 (40)	100 (8/8)	*ΔcrtMN* (Qxyl_Ec) knock-in at *aprE* site
DGI (*spoVG* + *amyE*)	pJOE895	138 ± 22	95 ± 15 (68.8 ± 2.8)	92.5 (40)	100 (18/18)	*ΔcrtMN* (Q_Ec) double knock-in at *spoVG* and *amyE* locus site
	pJOE896	38 ± 9	4 ± 3 (10 ± 8.8)	100 (12)	100 (12/12)	*ΔcrtMN* (Qxyl_Ec) triple knock-in at *spoVG* and *amyE* locus site
TGI (*spoVG* + *amyE* + *aprE*)	pJOE895	120 ± 19	6 ± 2 (4.9 ± 1.2)	94.4 (18)	100 (17/17)	*ΔcrtMN* (Q_Ec) triple knock-in at *spoVG, amyE* and *aprE* locus site
	pJOE896	35 ± 6	0	0	0	*ΔcrtMN* (Qxyl_Ec) triple knock-in at *spoVG, amyE* and *aprE* locus site

^a^Total number of transformants

^b^Clones were tested after three passages at 50 ºC

^c^Clones that showed PCR products out of total number of clones analyzed by colony PCR is depicted in parenthesis

^d^pJOE894 contains no repair template

^e^pJOE895 contains Q_Ec (2894-bp) as a repair template

^f^pJOE896 contains Qxyl_Ec (4476-bp) as a repair template

### Development, evaluation, and validation of novel CRISPR-Cas9 system for simultaneous insertion of multiple gene-copies

**Fig. 2:**
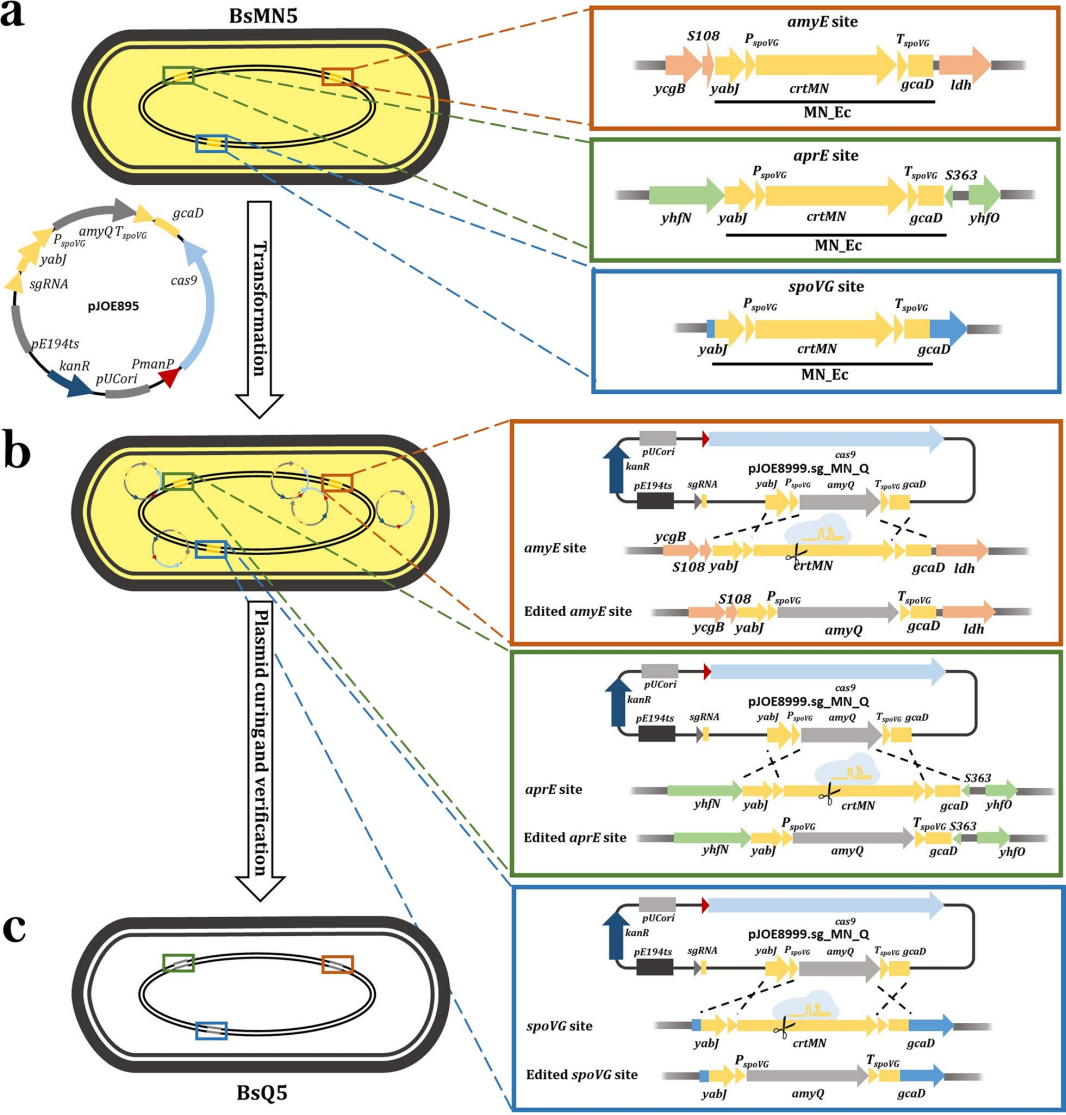
Outline of the proposed molecular mechanism of *crtMN* operon replacement for *amyQ* gene. **a** First step is to transform yellowish-pigmented BsMN5 strain with pJOE895 editing plasmid. Insets show MN_Ec integrated at *spoVG, amyE,* and *aprE* locus sites. **b** Upon transformation, the expression of Cas9 is guided by a specific sgRNA targeting multiple *crtMN* operon, thus generating DSBs at *spoVG*, *amyE* and *aprE* locus sites within BsMN5 chromosome. Insets depict homologous recombination with editing template provided by the plasmid, resulting in the replacement of three *crtMN* operon with three *amyQ* gene-copies, in a single step. Successful edited colonies (BsQ5) will appear as white clones, in contrast to unedited colonies that will remain yellow. **c** The final step is to verify the loss of the plasmid and the identity of the genome modification

To demonstrate the functionality of the CRSIPR-Cas9-based white/yellow screening method, we first constructed plasmid pJOE895. This plasmid was engineered to contain a homology repair expression cassette (Q_Ec) composed of the 530-bp homologous region upstream of *spoVG,* followed by a synthetic 1829-bp DNA fragment coding for amylase gene *amyQ* from *Bacillus amyloliquefaciens* and the 535-bp homologous region downstream of *spoVG*. We used an α-amylase as a secreted reporter system due to easy quantification of its extracellular production in production media. The rationale behind our procedure is that transformation of yellow-pigmented strains (BsMN1-BsMN5) with pJOE895 will allow the selective cleavage of the chromosome at specific sites with an integrated *crtMN* operon. After chromosome cleavage, homologous recombination with editing template will restore chromosome integrity and replace the *crtMN* operon with Q_Ec, thus leading to the formation of white colonies in successful edits (BsQ1–BsQ5). Conversely, unsuccessful genome edits will keep the *crtMN* operon intact, and the bacterial colonies will thus remain yellow. A scheme of the molecular mechanism proposed for the colorimetric detection of multiple *crtMN* operon replacement for *amyQ* gene is depicted in Fig. 2.

**Fig. 3:**
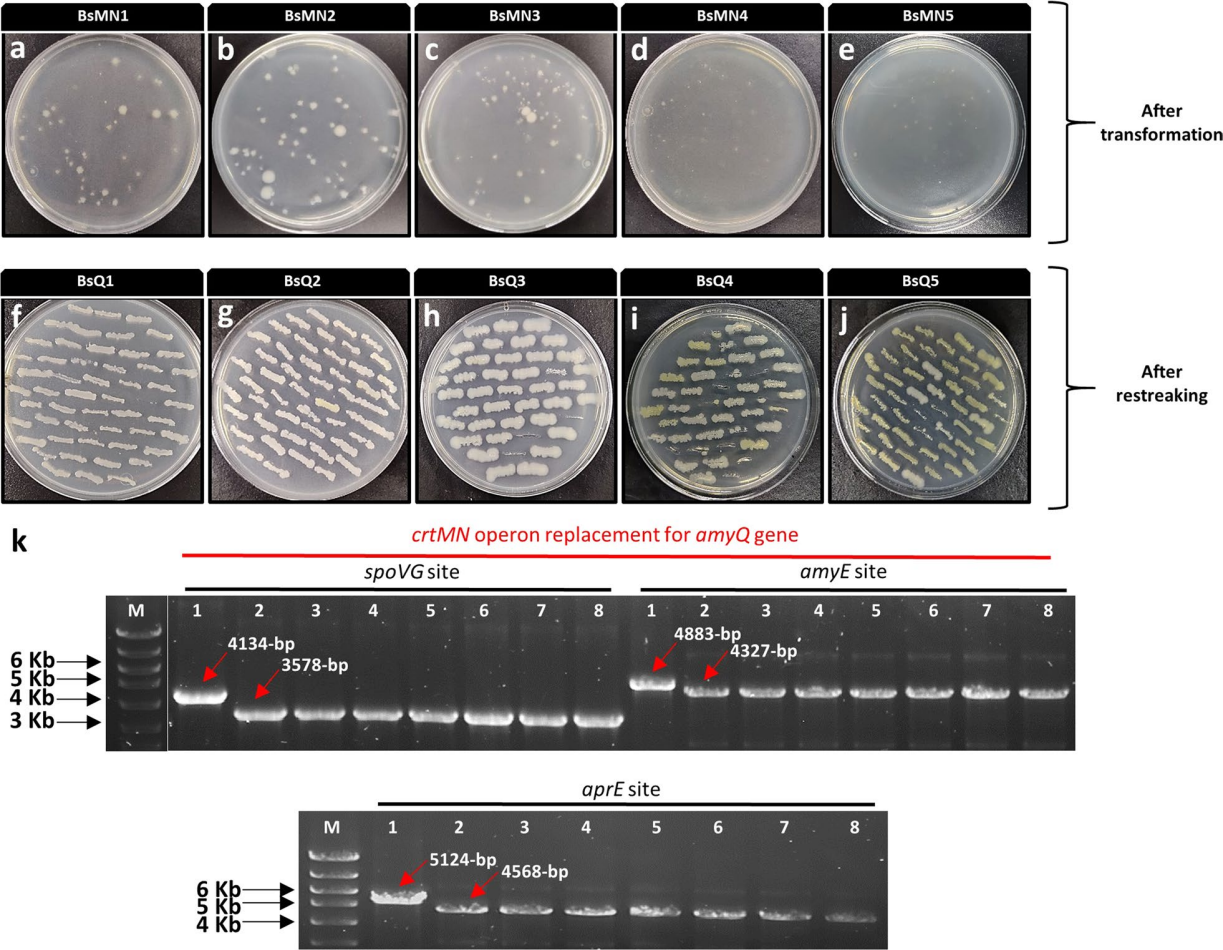
Transformation of pJOE895 plasmid and BsMN1-BsMN5-targeting plasmid. **a**–**e** Resulting colonies after transformation in LB plates with kanamycin. **f**–**j** Resulting clones after restreaking obtained transformants in LB plates with kanamycin and mannose. **k** PCR verification of randomly selected white clones derived from BsQ5 strain (as shown in panel j). The three *crtMN* operon replacements for *amyQ* gene were confirmed by visualization of correctly sized PCR products using P17F/P17R primers (*spoVG* site), P18F/P18R (*amyE* site) and P19F/P19R (*aprE* site), corresponding to lanes 2 to 8, which showed a reduction in size relative to control product with same primers (lane 1) at different locus sites. M corresponds to the molecular weight marker

**Fig. 4:**
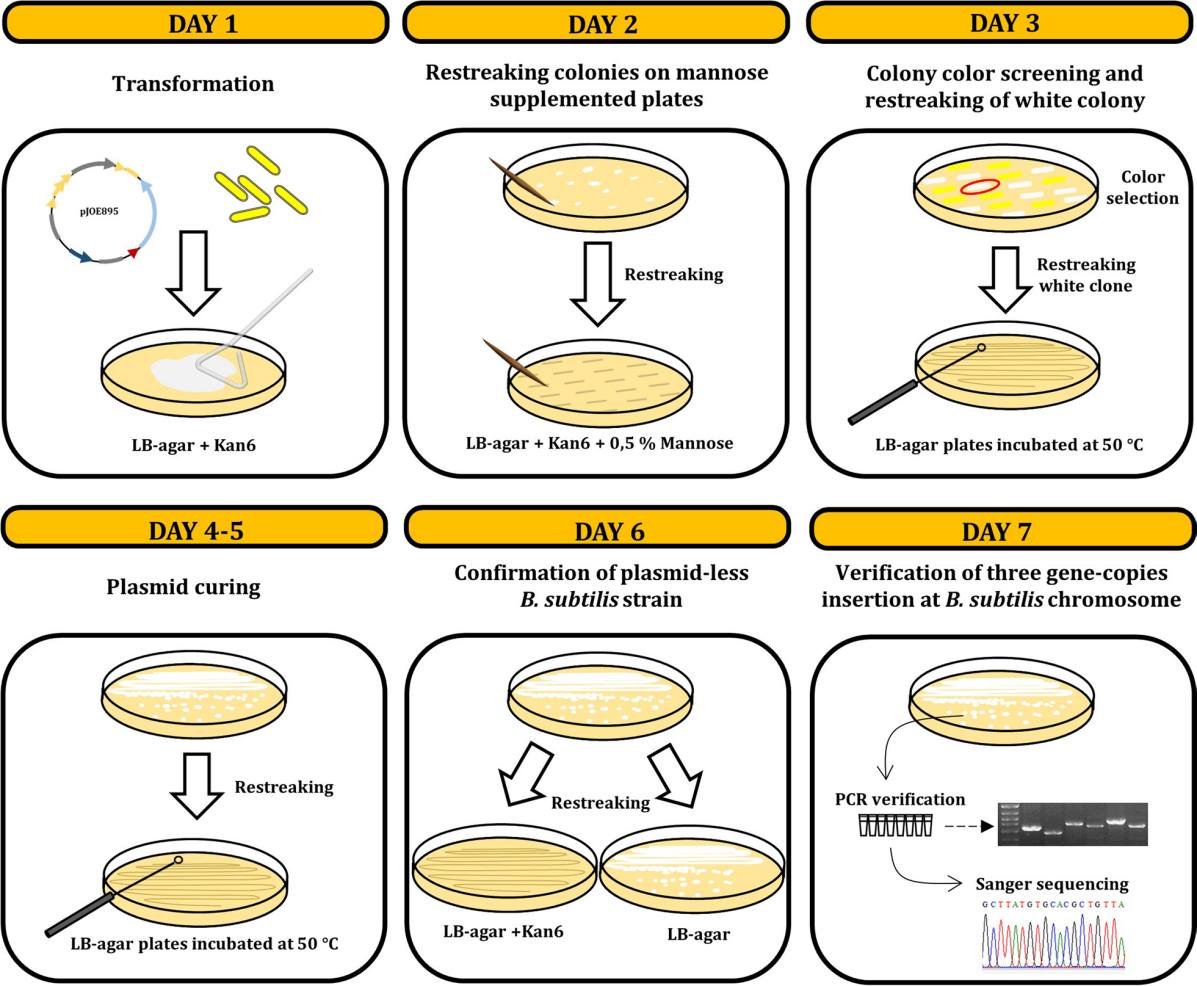
Schematic diagram of the white/yellow colorimetric screening method for the one-step multiple gene insertion detection in *B. subtilis* using the CRISPR-Cas9 system. Day 1: Transformation of *B. subtilis* with a single plasmid carrying a specific sgRNA targeting *crtMN* operon and desired editing template. Day 2: Transformants were spread on a LB plate containing kanamycin and mannose. Day 3: Resulting white clones were restreaked on LB-agar plates and incubated at 50 ºC. Day 4–5: White clones were cured from the plasmid after three passages at 50 ºC. Day 6: Colonies were replicated on LB plates with and without kanamycin. Day 7: Antibiotic sensitive colonies were then subjected to PCR verification and Sanger sequencing to verify their identities

Here, as a proof of principle, we tested the possibility of one-, two- and three-copy *amyQ* gene integration into the *B. subtilis* chromosome in a single step. For this application, BsMN1-BsMN5 strains were transformed with pJOE895 and resulting transformants were selected on LB plates supplemented with kanamycin (to select for the plasmid) at 37 ºC for 24 h. After incubation, the resultant colonies showed a filamentous aspect with irregular borders and without significant differences in their colony color (Fig. 3a–e). These filamentous colonies were immediately streaked on new LB plates with kanamycin and mannose and incubated at 37 ºC for an additional 24 h. The following day, we could readily distinguish between yellow and white bacterial clones in resulting strains (BsQ1–BsQ5) (Fig. 3f–j). Noticeably, some filamentous colonies derived from double- (DGI) and triple-*amyQ* gene integration (TGI) were unable to grow in the new plate. We then patched randomly selected white clones derived from each single *amyQ*-gene integration (SGI), DGI and TGI onto LB plates without antibiotics at 50 ºC to facilitate plasmid curing, which was achieved for most of the white clones tested after three passages at 50 ºC (Table 1). The plasmid-cured cells were checked by colony-PCR using the outer primers from the specific insertion sites to confirm the successful integrations. All white clones tested contained the desired replacements, as confirmed by checking the size of PCR fragments spanning the desired integration and by DNA sequencing (Fig. 3k). Overall, this meant it took around one week to construct a high-copy *B. subtilis* strain harboring three *amyQ* gene copies ready for characterization or another round of genetic manipulation (see Fig. 4). These results validate our procedure to discern between unedited yellow colonies from edited white colonies. Furthermore, the power and convenience of the high-throughput colorimetric screening method was duly proved, demonstrated by its ability for the rapid in vitro screening of large number of transformants and a dramatic decrease in the rate of false positives, allowing the identification of multigene insertion *B. subtilis* strains with 100% positivity among all clones tested (Table 1).

This procedure was also employed to monitor integration efficiency by simply counting colony color upon transformation. Therefore, integration efficiencies were systematically calculated as the ratio of the number of white colonies (correctly edited transformants) to the total number of transformants (white colonies plus yellow colonies). The simultaneous integration efficiency achieved for TGI was very low (4.9% ± 0.8%), significantly lower than efficiency achieved for DGI (68.8 ± 2.8%). In contrast, very high efficiencies that ranged from 97.5 to 98.6% for single *amyQ* gene integration (SGI) were achieved (Table 1). Moreover, CFUs were counted to analyze cell growth with no significant differences observed in the total number of transformants obtained from SGI, DGI, and TGI (Table 1). Gratifyingly, not only was the goal of inserting two- and three-gene copies into the *B. subtilis* chromosome in a single step accomplished for the first time by this novel methodology, but also the simple and fast identification of positive clones with desired genetic modifications.

### Expanding the genetic toolbox for promoter switching

**Fig. 5:**
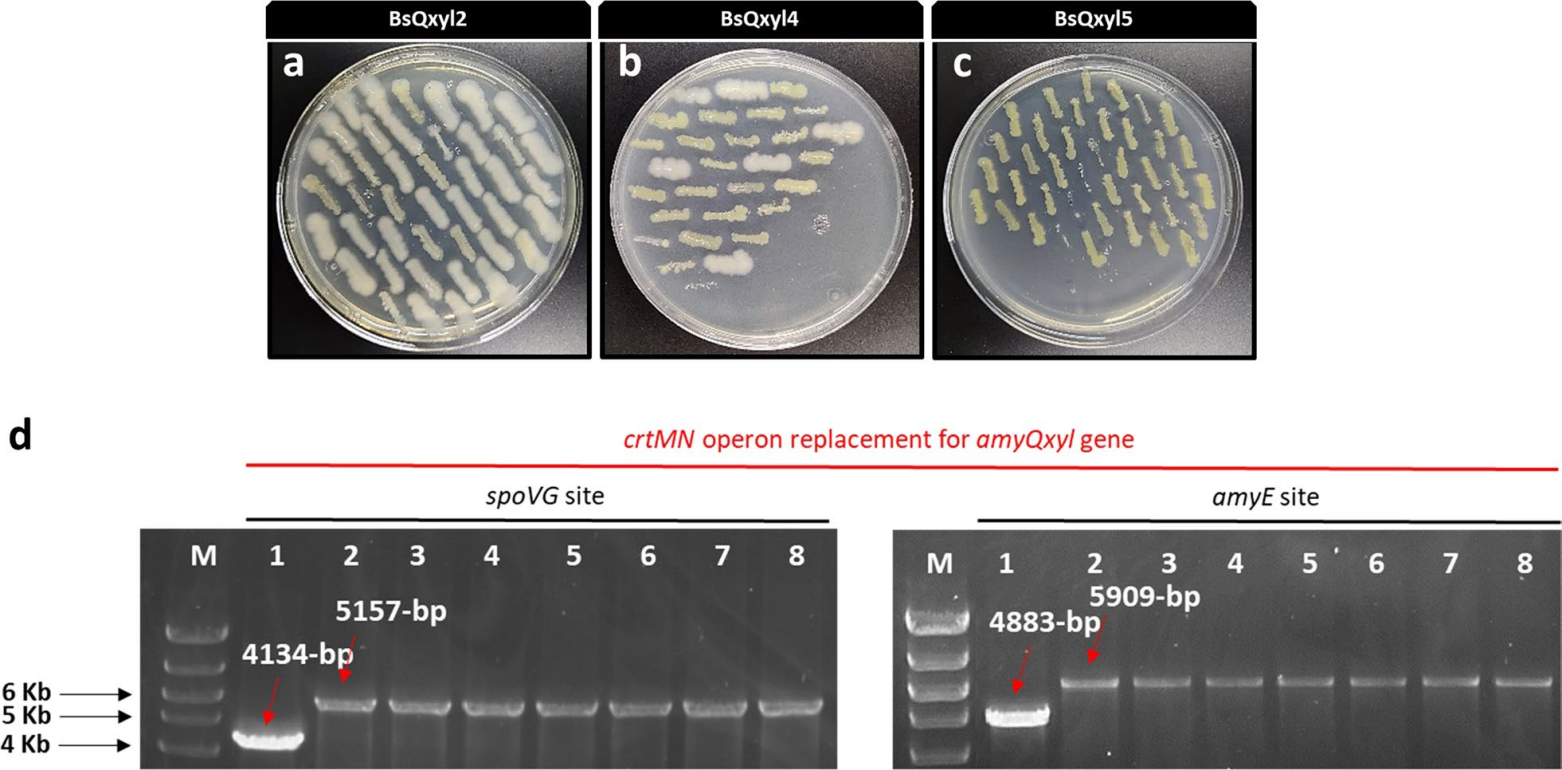
Transformation of BsMN2, BsMN4 and BsMN5 with pJOE896 plasmid. **a**–**c** Resulting white and yellow clones after transformation and restreaking of the obtained colonies in LB plates with kanamycin and mannose. **d** PCR verification of randomly selected white clones derived from BsQxyl4 strain (as shown in panel b). The two *crtMN* operon replacements for *amyQxyl* gene were confirmed by visualization of correctly sized PCR products using P17F/P17R primers (*spoVG* site) and P18F/P18R (*amyE* site) (lanes 2 to 8), which showed an increase in size relative to control product with same primers (lane 1) at different locus sites. M corresponds to the molecular weight marker

A key aspect of the novel editing strategy presented here is that a unique sgRNA along with changeable editing template would be sufficient for simultaneous targeting and insertion of multiple gene copies in *B. subtilis*. Hence, the flexibility of editing template construction opens the possibility of using this system to insert any gene under the control of desired promoters, which are indispensable control elements to accurately regulate expression of target proteins [41]. To begin exploring this application, we aimed to switch the P_*spoVG*_ controlling *amyQ* gene expression for the xylose-inducible promoter xylose-repressor system (P_*xyl*_). For this purpose, an engineered editing template (Qxyl_Ec) composed of 536-bp homologous upstream arm, followed by the 3405-bp *amyQxyl* gene (*amyQ* gene under the control of P_*xyl*_) and 535-bp homologous downstream arm, was constructed and inserted into the plasmid pJOE894 to yield pJOE896. We placed a stop codon at position + 6 (relative to *crtM* gene start codon) in the homologous upstream arm to prevent expression from the *spoVG* promoter. Upon transformation of yellow-pigmented strains with pJOE896, we were again able to recover white colonies from SGI and DGI integration experiments (Fig. 5a, b), although with very low efficiency for the double integrations (10% ± 8.8%; Table 1). In contrast, no white colonies for TGI were observed in three independent experiments (Fig. 5c, Table 1). After curing of the plasmid, all recovered white clones tested contained the desired Qxyl_Ec insertion, as verified by PCR amplification and Sanger sequencing (Fig. 5d), thus confirming the successful insertion of two Qxyl_Ec copies into the *B. subtilis* chromosome. Finally, we observed a marked decrease in the number of CFUs (35 to 61 CFU/μg DNA) in comparison to previous experiments with pJOE895 (120 to 150 CFU/μg DNA, Table 1).

### Monitoring amylase AmyQ secretion on liquid cultures

**Fig. 6:**
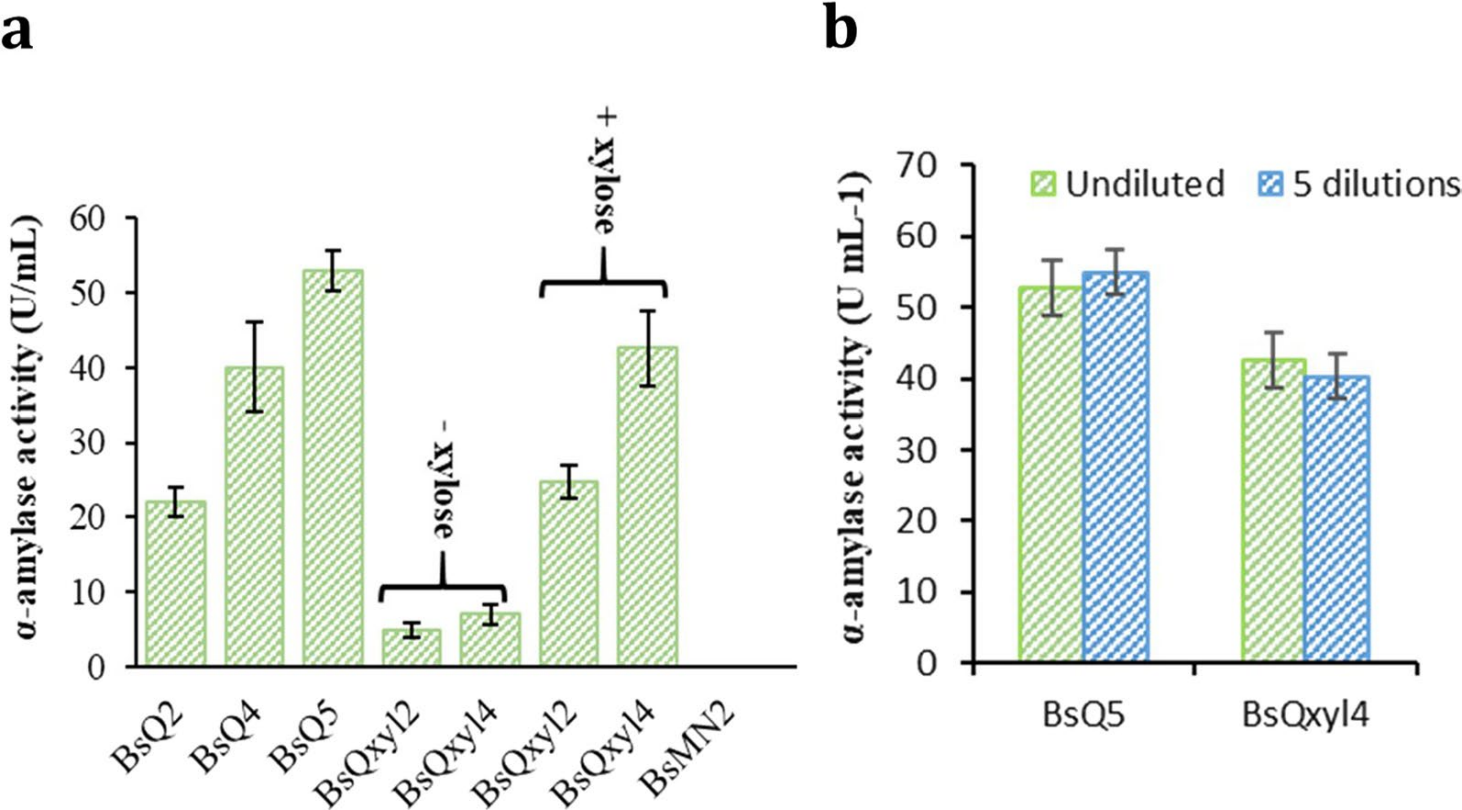
Detection of α-amylase activity in engineered B. subtilis strains. **a** α-amylase activity derived from supernatants of strains: BsQ2, BsQ4, BsQ5, BsQxyl2 and BsQxyl4. BsMN2 was used as a control strain. Experiments without (-xylose) and with xylose (+ xylose) are indicated. **b** α-amylase activity in supernatants from BsQ5 and BsQxyl4 and from the same strains diluted 1000-fold and grown to stationary phase repeatedly 5 times without antibiotics in LB media. The error bars represent the average ± standard deviation of three biological replicates

The effect of multiple Q_Ec and Qxyl_Ec copies inserted into the *B. subtilis* chromosome in the release of extracellular amylases was investigated by culturing recombinant strains in amylase production media and measuring the values of α-amylase activity secreted to the media. Strain BsQ2, which had a truncated copy of the *amyE* gene in the chromosome, rendering the strain inactive for amylase production, was used to evaluate the effect of one Q_Ec insertion and compared to BsQ4 and BsQ5 with two and three copies, respectively. Enzymatic assays showed that the higher the copy number of Q_Ec, the greater the values of α-amylase activity secreted to the media, achieving a value of 21.9 ± 2.1 U/ml in BsQ2, which was doubled in BsQ4 (40.2 ± 5.9 U/ml) and almost tripled in BsQ5 (53 ± 2.7 U/ml) (Fig. 6a). Although BsQxyl2 and BsQxyl4 showed low levels of α-amylase activity in production media (4.9 ± 0.9 U/ml and 7.0 ± 1.3 U/ml, respectively), seemingly resulting from basal promoter activity, α-amylase activity was highly induced with xylose: 24.7 ± 2.2 U/ml and 42.5 ± 4.8 U/ml, respectively (Fig. 6a), demonstrating the tight regulation of *amyQ* gene expression achieved through this system. Overall, these results confirm the dependence of extracellular amylase activity on *amyQ* gene copy-number and promoter type, thus highlighting the importance of multicopy strains for maximizing gene expression. Noticeably, we demonstrated that engineering the editing template could allow gene of interest expression with high precision.

The stability of strains BsQ5 and BsQxyl4 in the production of amylase AmyQ without antibiotic selection was tested. Overnight cultures of both strains in LB were diluted 1:1000 in the same medium. The cells were grown in shaking flasks at 37 °C to stationary phase and diluted again 1000-fold. This was repeated five times and in the last transfer, when the stationary phase was reached, strains were cultured in production media and α-amylase activity was determined. Figure 6b shows that both strains produced similar levels of α-amylase activity for at least 100 generations (every round of growth to stationary phase corresponds to about ten generations without antibiotic supplementation), demonstrating that this technology is a valuable tool for constructing stable producer *B. subtilis* strains.

## Discussion

Various high efficiency, simultaneous multilocus integration methods based on two-plasmid CRISPR-Cas9 systems have been implemented in *E. coli* [42, 43], yeasts [44, 45], and fungi [46, 47], making them suitable host cells to be used as a chassis in the field of synthetic biology. However, although the emergence of the CRISPR/Cas9 system has led to numerous new applications and developments in *B. subtilis* [29, 30, 48–51], efficient methods for simultaneous gene insertion in *B. subtilis* have not yet been reported. The increase of gene copy number and its integration into multiple neutral sites is the most promising way to achieve high yield and stable productivity, as a preliminary step for industrial strain development [16–18, 52, 53]. However, although this strategy is a valuable approach for the construction of multicopy *B. subtilis* strains, it relies on iterative rounds of plasmid construction for sequential insertions of gene copies into the *B. subtilis* chromosome, which is tedious and time consuming. This prompted us to investigate new methods to facilitate multigene insertions in a single step, therefore bypassing the big efforts needed for multi-step construction of plasmids and time-consuming curing of the plasmids in iterative genome editing.

The single-plasmid-based CRISPR-Cas9 system established in this study is preferred over genome engineering methods based on two-plasmid systems because of both the higher plasmid transformation efficiencies achieved and a marked decrease in the burden of the host strain [54]. This technology boasts two unique features that facilitate the simultaneous insertion of multiple gene copies into the *B. subtilis* chromosome. First, an invariable sgRNA was selected to reduce the off-target effects of endonuclease Cas9 and to ensure high specificity for the *crtMN* operon. Second, a changeable editing template containing the gene of interest flanked with homologous arms was demonstrated to be sufficient to replace the *crtMN* operon with the desired gene and allow white/yellow screening for successful insertions.

Although HDR efficiency using a single-plasmid system is greatly dependent on the length of the homologous repair template [51], our results corroborated that 530-bp homology arms were enough to allow multicopy integration with high efficiency. Additionally, we observed that the natural NHEJ system present in *B. subtilis* is too weak to repair DSBs in *B. subtilis*, which is in accordance with previous reports [51, 55]. Hence, as a proof of principle, the novel genome-editing method has been proven to be efficient for the simultaneous insertion of three 2894-bp, and two 4476-bp fragments containing Q_Ec and Qxyl_Ec, respectively, with Qxyl_Ec being the largest fragment reported to be integrated into the *B. subtilis* genome using the CRISPR-Cas9 system, so far.

The simultaneous insertion of three Qxyl_Ec copies into the *B. subtilis* genome was unsuccessful. We propose that the reason for failure might be associated with the size of the editing template. Qxyl_Ec is 4476-bp, which may be too large to efficiently initiate the double exchange, thus significantly lowering the probability for the insertion of more than one gene at a time. Therefore, for further practical application using this system, the size of the editing template should be carefully considered for expression cassettes longer than 3-kb.

Despite the very low efficiencies achieved for DGI and TGI, the colorimetric screening method adopted in this work represents a good solution to tackle the issue of low editing efficiencies achieved in multiplex genome editing, allowing the straightforward identification of positive clones with the desired genetic changes. Remarkably, this procedure resulted in saving time and cost and allowed the rapid and convenient construction of high-level expression of multicopy genes encoding secreted α-amylase in *B. subtilis* in only seven days.

Multilocus integration experiments resulted in the isolation of recombinant colonies showing a filamentous aspect, forming colonies with irregular borders and different sizes. The presence of this colony type may be related to the SOS response in *B. subtilis*. We suspect that the bacteria were edited during growth, thus causing some damage to the *B. subtilis* DNA which, as previously reported, triggers a physiological response called the SOS response [56], thus inhibiting cell division and causing bacteria to appear filamentous [33, 57–59]. Although most of the colonies grew normally after being subcultured on mannose plates, the growth of a few colonies was impaired, which irremediably led to cell death. We suspect that this phenomenon might be due to their inability to repair damaged DNA, which is supported by a similar observation recently reported by the group of Guo [33].

The versatility and wide range of applicability of the novel technology is considerable and was demonstrated in this study by engineering editing template to allow the replacement of constitutive P_*spoVG*_ for xylose-inducible P_*xyl*_. As one of the main difficulties in genetic engineering and synthetic biology is how to control the expression of a certain protein at a given level, our system provides an efficient, and facile approach for achieving desired production goals by selecting a suitable promoter element to accurately regulate expression of target proteins. For each application, the only requisite is to engineer the editing template to contain the desired cargo (gene of interest with or without modifications) flanked by 530-bp homologous arms to enable its multiple insertion under the control of desired promoter. In the case that higher levels of protein production are required, the use of dual and multiple tandem promoters has been shown to enhance productivity in *B. subtilis* [60–62], which can be also achieved by rational design of the editing template to contain the synthetic promoter. Conversely, if temporal control of gene expression is needed, the use of inducible promoters is of great interest, especially for the fine induction of difficult to express or toxic proteins [63]. Conclusively, editing template engineering strategies will allow fine-tuning of multicopy gene expression, which was successfully evaluated by means of a model protein, amylase AmyQ from *B. amyloliquefaciens* in this study. While the strategy presented here has been adopted for *B. subtilis*, we hope that this methodology will be applicable not only to other *Bacillus* species but to other relevant bacterial strains used in industrial applications, such as *Pseudomonas putida* and *Corynebacterium glutamicum*, among others [64]. Strains and plasmids developed in this work will be publicly available to the scientific community through the Bacillus Genetic Stock Center (https://bgsc.org/).

## Conclusions

Efficient multigene integration methods have not been developed in the model gram-positive *B. subtilis*, which is considered one of the dominant bacterial workhorses in microbial fermentation. For the first time, a simple, rapid, and convenient approach to facilitate the simultaneous insertion of up to three gene copies in *B. subtilis* was established through the development of a high-throughput colorimetric screening method combined with the use of a single CRISPR-Cas9-based plasmid carrying a unique high-efficiency target site and a changeable editing template with 530-bp homologous arms. Additionally, we demonstrated that the system could be adapted to modulate the promoter controlling the expression of the gene of interest by rational design of the editing template. This novel technology can be potentially used as a routine method for the construction of marker-free, plasmid-less, high-expression stable producer strains in a timely fashion, which provides promising prognoses for future developments of *B. subtilis* as a microbial cell factory in industrial settings.

## Methods

### Strains, media and growth conditions

*Escherichia coli* strain DH5α was used as the host strain for routine molecular cloning and plasmid construction operations. For transformation of *B. subtilis*, plasmid DNA was isolated from the rec^+^ strain *E. coli* Turbo (New England Biolabs). *B. subtilis* strain BsMN0, an asporogenous strain with seven protease genes inactivated, was purchased from the Bacillus Genetic Stock Center (BGSC, Ohio) and served as the recipient strain for the genome editing experiments. Chemically competent *E. coli* cells and transformation protocol were performed as described previously [65]. *B. subtilis* strains were transformed according to the method previously described [66]. The strains involved in this study are listed in Table 2. Strains were propagated in Lysogeny Broth (LB) medium (10 g/L tryptone, 5 g/L yeast extract, and 10 g/L NaCl) and on LB agar (15 g/L agar) plates at 37 °C. The plasmid used for the expression of the CRISPR–Cas9 constructs is based on vector pJOE8999 [27]. Kanamycin was used for screening in *E. coli* and *B. subtilis* at final concentrations of 30 µg/mL and 6 µg/mL, respectively. To induce the CRISPR-Cas9 system in *B. subtilis* cells, 0.5% D-mannose was added.

**Table 2:** Strains used in this study

Strain	Genotype or description	Source/Reference
*E. coli* DH5α	*fhuA2 lac(del)U169 phoA glnV44 Φ80' lacZ(del)M15 gyrA96 recA1 relA1 endA1 thi-1 hsdR17*	Laboratory stock
*E. coli* NEB® turbo	*F' proA* + *B* + *lacIq ∆lacZM15 / fhuA2 ∆(lac-proAB) glnV galK16 galE15 R(zgb-210::Tn10)*TetS *endA1 thi-1 ∆(hsdS-mcrB)5*	Laboratory stock
*B. subtilis* strains		
BsMN0	*ΔnprE ΔaprE Δepr Δmpr ΔnprB Δvpr Δbpr ΔsigF*	Laboratory stock
BsMN1	BsMN0 with *ΔspoVG* (MN_Ec) knock-in mutant	This study
BsMN2	BsMN0 with *ΔamyE *(MN_Ec) knock-in mutant	This study
BsMN3	BsMN0 with *ΔaprE* (MN_Ec) knock-in mutant	This study
BsMN4	BsMN0 with *ΔspoVG ΔamyE* (MN_Ec) double knock-in mutant	This study
BsMN5	BsMN0 with *ΔspoVG ΔamyE ΔaprE* (MN_Ec) triple knock-in mutant	This study
BsQ1	BsMN1 with *Δ*MN_Ec (Q_Ec) knock-in mutant	This study
BsQ2	BsMN2 with *Δ*MN_Ec (Q_Ec) knock-in mutant	This study
BsQ3	BsMN3 with *ΔMN_Ec* (*Q*_Ec) knock-in mutant	This study
BsQ4	BsMN4 with* Δ*MN_Ec (Q_Ec) double knock-in mutant	This study
BsQ5	BsMN5 with* Δ*MN_Ec (Q_Ec) triple knock-in mutant	This study
BsQxyl2	BsMN2 with* Δ*MN_Ec* (*Qxyl_Ec) knock-in mutant	This study
BsQxyl4	BsMN4 with* Δ*MN_Ec (Qxyl_Ec) double knock-in mutant	This study

### Plasmid construction

The plasmids and primers used in this study are listed in Additional file 2: Tables S1 and S2, respectively. All plasmids construction were performed using the pJOE8999 plasmid as the parental plasmid and required two consecutive steps: (i) cloning specific sgRNA; and (ii) cloning specific editing template.

### Cloning of sgRNA

For sgRNA construction targeting each specific gene, two complementary oligonucleotides were ordered (Macrogen, Korea) with the respective overhangs, annealed and cloned into the vector pJOE8999. In brief, both complementary oligonucleotides were mixed at a final concentration of 10 µM in annealing buffer (10 × stock contained 100 mM Tris-HCl pH 7.5, 1 M NaCl and 1 mM Ethylenediaminetetraacetic acid (EDTA) (pH 8), kept at 98 °C for 5 min and slowly cooled to room temperature. Then, the annealed oligonucleotides were treated with polynucleotide kinase to phosphorylate the 5’ ends, according to manufacturer’s instructions (Invitrogen), and ligated to *BsaI* cleaved and dephosphorylated plasmid pJOE8999 to incorporate specific target sequences to the vector.

### Cloning of editing template

In a second step, to construct editing templates, two homologous arms of similar length and the desired cargo to be inserted were separately amplified and were then fused together by splicing with overlap extension PCR (SOEing-PCR, Additional file 2: Table S3). Regarding the desired cargo, the *amyQ* gene was synthesized by Nzytech (Portugal) with codon optimization for *B. subtilis* (Additional file 1: Fig. S1), and cloned into pBS2EXxylRPxylA vector by using restriction sites *XbaI* and *PstI*, resulting in the plasmid pBS2EXxylRPxylA_Q. This plasmid was used as a template for *amyQ* and *amyQxyl* gene amplifications, whereas pHY_crtMN vector provided by the Maeda’s group [37], was used as a template for *crtMN* operon amplification. SOEing-PCR products of the expected size along with pJOE8999 plasmids harboring specific target sequence were digested with *SfiI* and ligated with T4 DNA-ligase (Nzytech) to incorporate the desired cargo to the corresponding editing plasmid. All plasmids were verified by Sanger sequencing. A more detailed explanation for the construction of each editing plasmid is given in the supplementary material (Additional file 1: Fig. S2–S6).

### Construction of multicopy strains and plasmid curing

After transformation of *B. subtilis* strains with editing plasmids, resulting colonies obtained on LB agar plates with kanamycin were streaked into LB agar plates supplemented with kanamycin and 0.5% of D-mannose and incubated at 37 °C for 24 h. After incubation, white colonies were selected among yellow colonies and passaged three times on LB agar plates (without any antibiotics) at 50 °C for 24 h to cure the plasmid. Plasmid curing achieved best results when cells are streaked for single colonies at each passage. The colonies cured of editing plasmid were confirmed by streaking them onto LB agar plates containing kanamycin or no antibiotics; colonies cured of plasmid fail to grow at 37 °C. The presence of multiple gene copies was verified by colony PCR using relevant primers and Sanger sequencing (Additional file 2: Table S3).

### Quantification of α-amylase activity in liquid cultures

Overnight cultures of recombinant *B. subtilis* strains in production medium (12 g/L sucrose, 18 g/L peptone, 2 g/L (NH_4_)_2_SO_4_, 18.3 g/L K_2_HPO_4_·3H_2_O, 6 g/L KH_2_PO_4_, 1 g/L Na^+^ citrate, 0.2 g/L MgSO_4_·7H_2_O, 0.12 g/L FeSO_4_·7H_2_O, 30 mg/L MnSO_4_·H_2_O, 12 mg/L CuSO_4_·H_2_O and 12 mg/L ZnCl_2_) were diluted to 0.1 OD_600_ in 25 mL of production media and were grown at 37 ºC and 220 rpm for 2 days. In the case of xylose-inducing experiments, 1% xylose was added at OD_600_ between 0.6 and 0.8 and strains were then cultured for additional 48 h at same conditions. After 48 h, the culture supernatants were obtained by centrifugation at 8000 g for 20 min at 4 °C and were used as crude α-amylase samples. The standard assay mixture contained 1.5% soluble starch in a final volume of 0.25 ml of 50 mM Tris–HCl buffer at pH 6.5. The mixture was assayed with 2 μl of the α-amylase solution and incubated at 80 ºC for 2 min. Next, the reaction was stopped by the addition of 0.75 mL of 3,5- dinitrosalicylic acid (DNS) reagent then heated for 5 min in boiling water bath and cooled on ice. The absorbance was read at 540 nm and compared to a standard calibration curve for maltose (Additional file 1: Fig. S7). One unit of amylase activity was defined as the amount of enzyme which liberated 1 µmol of maltose from soluble starch per minute under the assay conditions [67].

## Supplementary Information

**Additional file 1: Figure S1.** *amyQ* gene sequence with codon optimization for *B. subtilis*. **Figure S2.** Construction system of vector pJOE891. **Figure S3.** Construction system of vector pJOE892. **Figure S4.** Construction system of vector pJOE893. **Figure S5.** Construction system of vector pJOE894 and pJOE895. **Figure S6.** Construction system of vector pJOE896.** Figure S7.** Standard calibration curve for maltose.

**Additional file 2: Table S1.** Plasmids used in this study. **Table S2.** Primers designed in this study. **Table S3.** Splicing with overlap extension PCR (SOEing-PCR) program.

## Data Availability

All data supporting the conclusions of this study are included within the article and its additional files.

## References

[1] Earl AM, Losick R, Kolter R (2008). Ecology and genomics of *Bacillus subtilis*. Trends Microbiol.

[2] Schallmey M, Singh A, Ward OP (2004). Developments in the use of *Bacillus* species for industrial production. Can J Microbiol.

[3] Outtrup H, Jorgensen ST (2008). The importance of *Bacillus* species in the production of industrial enzymes. Applications and systematics of *Bacillus* and relatives.

[4] Dijl JMV, Hecker M (2013). *Bacillus subtilis*: from soil bacterium to super-secreting cell factory. Microb Cell Fact.

[5] Liu DY, Mao ZT, Guo JX, Wei LY, Ma HW, Tang YJ (2018). Construction, model-based analysis, and characterization of a promoter library for fine-tuned gene expression in *Bacillus subtilis*. ACS Synth Biol.

[6] Danilova I, Sharipova M (2020). The practical potential of *Bacilli* and their enzymes for industrial production. Front Microbiol.

[7] Su Y, Liu C, Fang H, Zhang D (2020). *Bacillus subtilis*: a universal cell factory for industry, agriculture, biomaterials and medicine. Microb Cell Fact.

[8] Young M (1984). Gene amplification in *Bacillus subtilis*. J Gen Microbiol.

[9] Schumann W (2007). Production of recombinant proteins in *Bacillus subtilis*. Adv Appl Microbiol.

[10] Piggot PJ, Curtiss CAM (1987). Analysis of the regulation of gene expression during *Bacillus subitlis* sporulation by manipulation of the copy number of *spo-lacZ* fusions. J Bacteriol.

[11] Vazquez-Cruz C, Ochoa-Sanchez J, Olmedo-Alvarez G (1996). Pulse-field gel-electrophoretic analysis of the amplification and copy-number stability of an integrational plasmid in *Bacillus subtilis*. Appl Microbiol Biotechnol.

[12] Jannière L, Niaudet B, Pierre E, Ehrlich SD (1985). Stable gene amplification in the chromosome of *Bacillus subtilis*. Gene.

[13] Leenhouts KJ, Kok J, Venema G (1990). Stability of integrated plasmids in the chromosome of *Lactococcus lactis*. Appl Environ Microbiol.

[14] van der Laan JC, Gerritse G, Mulleners LJ, van der Hoek RA, Quax WJ (1991). Cloning, characterization, and multiple chromosomal integration of a *Bacillus* alkaline protease gene. Appl Environ Microbiol.

[15] Petit MA, Joliff G, Mesas JM, Klier A, Rapoport G, Ehrlich SD (1990). Hypersecretion of a cellulase from clostridium thermocellum in *Bacillus subtilis* by induction of chromosomal DNA amplification. Biotechnology (NY).

[16] Yomantas YA, Abalakina EG, Golubeva LI, Gorbacheva LY, Mashko SV (2011). Overproduction of *Bacillus amyloliquefaciens* extracellular glutamyl-endopeptidase as a result of ectopic multi-copy insertion of an efficiently expressed mpr gene into the *Bacillus subtilis* chromosome. Microb Cell Fact.

[17] Huang K, Zhang T, Jiang B, Yan X, Mu W, Miao M (2017). Overproduction of *Rummeliibacillus pycnus* arginase with multi-copy insertion of the *argR-pyc* cassette into the Bacillus subtilis chromosome. Appl Microbiol Biotechnol.

[18] Wang J-J, Rojanatavorn K, Shih JCH (2004). Increased production of *Bacillus* keratinase by chromosomal integration of multiple copies of the *kerA* gene. Biotechnol Bioeng.

[19] Zhang XZ, Yan X, Cui ZL, Hong Q, Li SP (2006). *mazF*, a novel counter-selectable marker for unmarked chromosomal manipulation in *Bacillus subtilis*. Nucleic Acids Res.

[20] Brans A, Filee P, Chevigne A, Claessens A, Joris B (2004). New integrative method to generate *Bacillus subtilis* recombinant strains free of selection markers. Appl Environ Microbiol.

[21] Defoor E, Kryger MB, Martinussen J (2007). The orotate transporter encoded by *oroP* from *Lactococcus lactis* is required for orotate utilization and has utility as a food-grade selectable marker. Microbiology.

[22] Fabret C, Ehrlich SD, Noirot P (2002). A new mutation delivery system for genome-scale approaches in *Bacillus subtilis*. Mol Microbiol.

[23] Shi T, Wang G, Wang Z, Fu J, Chen T, Zhao X (2013). Establishment of a markerless mutation delivery system in *Bacillus subtilis* stimulated by a double-strand break in the chromosome. PLoS ONE.

[24] Yan X, Yu HJ, Hong Q, Li SP (2008). Cre/lox system and PCR-based genome engineering in *Bacillus subtilis*. Appl Environ Microbiol.

[25] Chen PT, Jeifu S, Chao YP, Ho T, Yu SM (2010). Construction of chromosomally located T7 expression system for production of heterologous secreted proteins in *Bacillus subtilis*. J Agric Food Chem.

[26] Wang Y, Weng J, Waseem R, Yin X, Zhang R, Shen Q (2012). *Bacillus subtilis* genome editing using ssDNA with short homology regions. Nucleic Acids Res.

[27] Altenbuchner J (2016). Editing of the *Bacillus subtilis* genome by the CRISPRCas9 system. Appl Environ Microbiol.

[28] Burby PE, Simmons LA (2017). CRISPR/Cas9 editing of the bacillus subtilis genome. Bio Protoc.

[29] So Y, Park SY, Park EH, Park SH, Kim EJ, Pan JG (2017). A highly efficient CRISPR-Cas9-mediated large genomic deletion in *Bacillus subtilis*. Front Microbiol.

[30] Westbrook AW, Moo-Young M, Chou CP (2016). Development of a CRISPR-Cas9 tool kit for comprehensive engineering of *Bacillus subtilis*. Appl Environ Microbiol.

[31] Lu Z, Yang S, Yuan X, Shi Y, Ouyang L, Jiang S (2019). CRISPRassisted multi-dimensional regulation for fine-tuning gene expression in *Bacillus subtilis*. Nucleic Acids Res.

[32] Price MA, Cruz R, Baxter S, Escalettes F, Rosser SJ (2019). CRISPR-Cas9 In Situ engineering of subtilisin E in *Bacillus subtilis*. PLoS ONE.

[33] Tian J, Xing B, Li M, Xu C, Huo YX, Guo S (2022). Efficient large-scale and scarless genome engineering enables the construction and screening of *Bacillus subtilis* biofuel overproducers. Int J Mol Sci.

[34] Liu D, Huang C, Guo J, Zhang P, Chen T, Wang Z (2019). Development and characterization of a CRISPR/Cas9n-based multiplex genome editing system for *Bacillus subtilis*. Biotechnol Biofuels.

[35] Wu Y, Liu Y, Lv X, Li J, Du G, Liu L (2020). CAMERS-B: CRISPR/Cpf1 assisted multiple-genes editing and regulation system for *Bacillus subtilis*. Biotechnol Bioeng.

[36] Pelz A, Wieland KP, Putzbach K, Hentschel P, Albert K, Götz F (2005). Structure and biosynthesis of staphyloxanthin from *Staphylococcus aureus*. J Biol Chem.

[37] Yoshida K, Ueda S, Maeda I (2009). Carotenoid production in *Bacillus subtilis* achieved by metabolic engineering. Biotechnol Lett.

[38] Banner CD, Moran CP, Losick R (1983). Deletion analysis of a complex promoter for a developmentally regulated gene from *Bacillus subtilis*. J Mol Biol.

[39] Weller GR, Kysela B, Roy R, Tonkin LM, Scanlan E, Della M (2002). Identification of a DNA nonhomologous end-joining complex in bacteria. Science.

[40] Doench JG, Fusi N, Sullender M, Hegde M, Vaimberg EW, Donovan KF (2016). Optimized sgRNA design to maximize activity and minimize off-target effects of CRISPR-Cas9. Nat Biotechnol.

[41] Fu G, Yue J, Li D, Li Y, Lee SY, Zhang D (2022). An operator-based expression toolkit for *Bacillus subtilis* enables fine-tuning of gene expression and biosynthetic pathway regulation. Proc Natl Acad Sci.

[42] Zhu X, Zhao D, Qiu H, Fan F, Man S, Bi C (2017). The CRISPR/Cas9-facilitated multiplex pathway optimization (CFPO) technique and its application to improve the *Escherichia coli* xylose utilization pathway. Metab Eng.

[43] Jiang Y, Chen B, Duan C, Sun B, Yang J, Yang S (2015). Multigene editing in the *Escherichia coli* genome via the CRISPR-Cas9 system. Appl Environ Microbiol.

[44] Horwitz AA, Walter JM, Schubert MG, Kung SH, Hawkins K, Platt DM (2015). Efficient multiplexed integration of synergistic alleles and metabolic pathways in yeasts via CRISPR–Cas. Cell Syst.

[45] Wang L, Deng A, Zhang Y, Liu S, Liang Y, Bai H (2018). Efficient CRISPR–Cas9 mediated multiplex genome editing in yeasts. Biotechnol Biofuels.

[46] Katayama T, Nakamura H, Zhang Y, Pascal A, Fujii W, Maruyama JI (2019). Forced recycling of an AMA1-based genome-editing plasmid allows for efficient multiple gene deletion/integration in the industrial filamentous fungus *Aspergillus oryzae*. Appl Environ Microbiol.

[47] Pohl C, Kiel J, Driessen AJM, Bovenberg RAL, Nygard Y (2016). CRISPR/Cas9 based genome editing of *Penicillium chrysogenum*. ACS Synth Biol.

[48] Song Y, He S, Abdallah II, Jopkiewicz A, Setroikromo R, van Merkerk R (2021). Engineering of multiple modules to improve amorphadiene production in *Bacillus Subtilis* using CRISPR-Cas9. J Agric Food Chem.

[49] Wu G, Drufva E, Wu K (2019). Fast genome editing in *Bacillus subtilis*. Eng Life Sci.

[50] Sachla AJ, Alfonso AJ, Helmann JD (2021). A simplified method for CRISPR-Cas9 engineering of *Bacillus subtilis*. Microbiol Spectr.

[51] Toymentseva AA, Altenbuchner J (2019). New CRISPR-Cas9 vectors for genetic modifications of *Bacillus* species. FEMS Microbiol Lett.

[52] Watzlawick H, Altenbuchner J (2019). Multiple integration of the gene *ganA* into the *Bacillus subtilis* chromosome for enhanced β-galactosidase production using the CRISPR/Cas9 system. AMB Express.

[53] Widner B, Thomas M, Sternberg D, Lammon D, Behr R, Sloma A (2000). Development of marker-free strains of *Bacillus subtilis* capable of secreting high levels of industrial enzymes. J Ind Microbiol Biotech.

[54] Hong KQ, Liu DY, Chen T, Wang Z-W (2018). Recent advances in Crispr/Cas9 mediated genome editing in *Bacillus subtilis*. World J Microbiol Biotechnol.

[55] Zheng X, Li SY, Zhao GP, Wang J (2017). An efficient system for deletion of large DNA fragments in *Escherichia coli* Via introduction of both Cas9 and the non-homologous end joining system from *Mycobacterium smegmatis*. Biochem Biophys Res Commun.

[56] Au N, Kuester-Schoeck E, Mandava V, Bothwell LE, Canny SP, Chachu K, Colavito SA, Fuller SN, Groban ES, Hensley LA, O'Brien TC, Shah A, Tierney JT, Tomm LL, O'Gara TM, Goranov AI, Grossman AD, Lovett CM (2005). Genetic composition of the *Bacillus subtilis* SOS system. J Bacteriol.

[57] Kawai Y, Moriya S, Ogasawara N (2003). Identification of a protein, Ynea, responsible for cell division suppression during the SOS response in *Bacillus subtilis*. Mol Microbiol.

[58] Claessen D, Emmins R, Hamoen LW, Daniel RA, Errington J, Edwards DH (2008). Control of the cell elongation-division cycle by shuttling of Pbp1 protein in *Bacillus subtilis*. Mol Microbiol.

[59] Dion MF, Kapoor M, Sun Y, Wilson S, Ryan J, Vigouroux A (2019). *Bacillus Subtilis* cell diameter is determined by the opposing actions of two distinct cell wall synthetic systems. Nat Microbiol.

[60] Song Y, Nikoloff JM, Fu G, Chen J, Li Q, Xie N (2016). Promoter screening from *Bacillus subtilis* in various conditions hunting for synthetic biology and industrial applications. PLoS ONE.

[61] Liu X, Wang H, Wang B, Pan L (2018). Efficient production of extracellular pullulanase in *Bacillus subtilis* ATCC6051 using the host strain construction and promoter optimization expression system. Microb Cell Fact.

[62] Liu Z, Zheng W, Ge C, Cui W, Zhou L, Zhou Z (2019). High-level extracellular production of recombinant nattokinase in *Bacillus subtilis* WB800 by multiple tandem promoters. BMC Microbiol.

[63] Saccardo P, Corchero JL, Ferrer-Miralles N (2016). Tools to cope with difficult-to-express proteins. Appl Microbiol Biotechnol.

[64] Regassa H, Bose D, Mukherjee A (2021). Review of microorganisms and their enzymatic products for industrial bioprocesses. Ind Biotechnol.

[65] Chang AY, Chau VWY, Landas JA, Pang Y (2017). Preparation of calcium competent *Escherichia coli* and heat-shock transformation. JEMI methods.

[66] Yasbin RE, Wilson GA, Young FE (1975). Transformation and transfection in lysogenic strains of *Bacillus subtilis*: evidence for selective induction of prophage in competent cells. J Bacteriol.

[67] Yang H, Liu L, Li J, Du G, Chen J (2011). Heterologous expression, biochemical characterization, and overproduction of alkaline α-amylase from *Bacillus alcalophilus* in *Bacillus subtilis*. Microb Cell Fact.

