# Supplementary material for: Barriers to simultaneous multilocus integration in Bacillus subtilis tumble down: development of a straightforward screening method for the colorimetric detection of one-step multiple gene insertion using the CRISPR-Cas9 system
Source: Microb Cell Fact. 2023 Jan 31;22:21. doi: 10.1186/s12934-023-02032-2 (PMC9890709; doi:10.1186/s12934-023-02032-2)
Supplement: Supplementary file 1 — Additional file 1: Figure S1. amyQ gene sequence with codon optimization for B. subtilis. Figure S2. Construction system of vector pJOE891. Figure S3. Construction system of vector pJOE892. Figure S4. Construction system of vector pJOE893. Figure S5. Construction system of vector pJOE894 and pJOE895. Figure S6. Construction system of vector pJOE896. Figure S7. Standard calibration curve for maltose. [file 12934_2023_2032_MOESM1_ESM.docx]

**Barriers to simultaneous multilocus integration in *Bacillus subtilis* tumble down: development of a straightforward screening method for the colorimetric detection of one-step multiple gene insertion using the CRISPR-Cas9 system**

Jordi Ferrando, Oriana Filluelo, Daniel R Zeigler and Pere Picart

**SUPPLEMENTARY MATERIAL**

**Additional file 1**

**Figure S1**: *amyQ* gene sequence with codon optimization for *B. subtilis*

**Figure S2**: Construction system of vector pJOE891

**Figure S3**: Construction system of vector pJOE892

**Figure S4**: Construction system of vector pJOE893

**Figure S5**: Construction system of vector pJOE894 and pJOE895

**Figure S6**: Construction system of vector pJOE896

**Figure S7**: Standard calibration curve for maltose

TAAGAAAG**GGAGG**ACAAACATGATTCAAAAACGAAAGCGGACAGTTTCGTTCAGACTTGTGCTTATGTGCACGCTGTTATTTGTCAGTTTGCCGATTACAAAAACATCAGCCGTTAACGGCACACTTATGCAATACTTCGAATGGTACACACCTAACGATGGCCAACATTGGAAACGTCTTCAAAACGATGCTGAACATCTTTCTGATATCGGCATCACAGCTGTTTGGATACCTCCTGCTTACAAAGGCCTTTCTCAATCTGATAACGGCTACGGCCCTTACGATCTTTACGATCTTGGCGAATTCCAACAAAAAGGCACAGTTCGTACAAAATACGGCACAAAATCTGAACTTCAAGATGCTATCGGCTCTCTTCATTCTCGTAACGTTCAAGTTTACGGCGATGTTGTTCTTAACCATAAAGCTGGCGCTGATGCTACAGAAGATGTTACAGCTGTTGAAGTTAACCCTGCTAACCGTAACCAAGAAACATCTGAAGAATACCAAATCAAAGCTTGGACAGATTTCCGTTTCCCTGGCCGTGGCAACACATACTCTGATTTCAAATGGCATTGGTACCATTTCGATGGCGCTGATTGGGATGAATCTCGTAAAATCTCTCGTATCTTCAAATTCCGTGGCGAAGGCAAAGCTTGGGATTGGGAAGTTTCTTCTGAAAACGGCAACTACGATTACCTTATGTACGTTGATGTTGATTACGATCATCCTGATGTTGTTGCTGAAACAAAAAAATGGGGCATCTGGTACGCTAACGAACTTTCTCTTGATGGCTTCCGTATCGATGCTGCTAAACATATCAAATTCTCTTTCCTTCGTGATTGGGTTCAAGCTGTTCGTCAAGCTACAGGCAAAGAAATGTTCACAGTTGCTGAATACTGGCAAAACGATGCTGGCAAACTTGAAAACTACCTTAACAAAACATCTTTCAACCAATCTGTTTTCGATGTTCCTCTTCATTTCAACCTTCAAGCTGCTTCTTCTCAAGGCGGCGGCTACGATATGCGTCGTCTTCTTGATGGCACAGTTGTTTCTCGTCATCCTGAAAAAGCTGTTACATTCGTTGAAAACCATGATACACAACCTGGCCAATCTCTTGAATCTACAGTTCAAACATGGTTCAAACCTCTTGCTTACGCTTTCATCCTTACACGTGAATCTGGCTACCCTCAAGTTTTCTACGGCGATATGTACGGCACAAAAGGCACATCTCCTCGTGAAATCCCTTCTCTTAAAGATTCTATCGAACCTATCCTTAAAGCTCGTAAAGAATACGCTTACGGCCCTCAACATGATTACATCGATCATCCTGATGTTATCGGCTGGACACGTGAAGGCGATTCTTCTGCTGCTAAATCTGGCCTTGCTGCTCTTATCACAGATGGCCCTGGCGGCTCTAAACGTATGTACGCTGGCCTTAAAAACGCTGGCGAAACATGGTACGATATCACAGGCAACCGTTCTGATACAGTTAAAATCGGCTCTGATGGCTGGGGCGAATTCCATGTTAACGATGGCTCTGTTTCTATCTACGTTCAAAAATAAGGTAATAAAAAAACACCTCCAAGCTGAGTGCGGGTATCAGCTTGGAGGTGCGTTTATTTTTTCAGCCGTATGACAAGGTCGGCATCAGGTGTGACAAATACGGTATGCTGGCTGTCATAGGTGACAAATCCGGGTTTTGCGCCGTTTGGCTTTTTCACATGTCTGATTTTTGTATAATCAACAGGCACGGAGCCGGAATCTTTCGCCTTGGAAAAATAAGCGGCGATCGTAGCTGCTTCCAATATGGATTGTTCATCGGGATCGCTGCTTTTAATCACAACGTGA

**Figure S1.** *amyQ* gene sequence with codon optimization for *B. subtilis*. The ribosomal binding site is marked in bold and the signal peptide is underline.


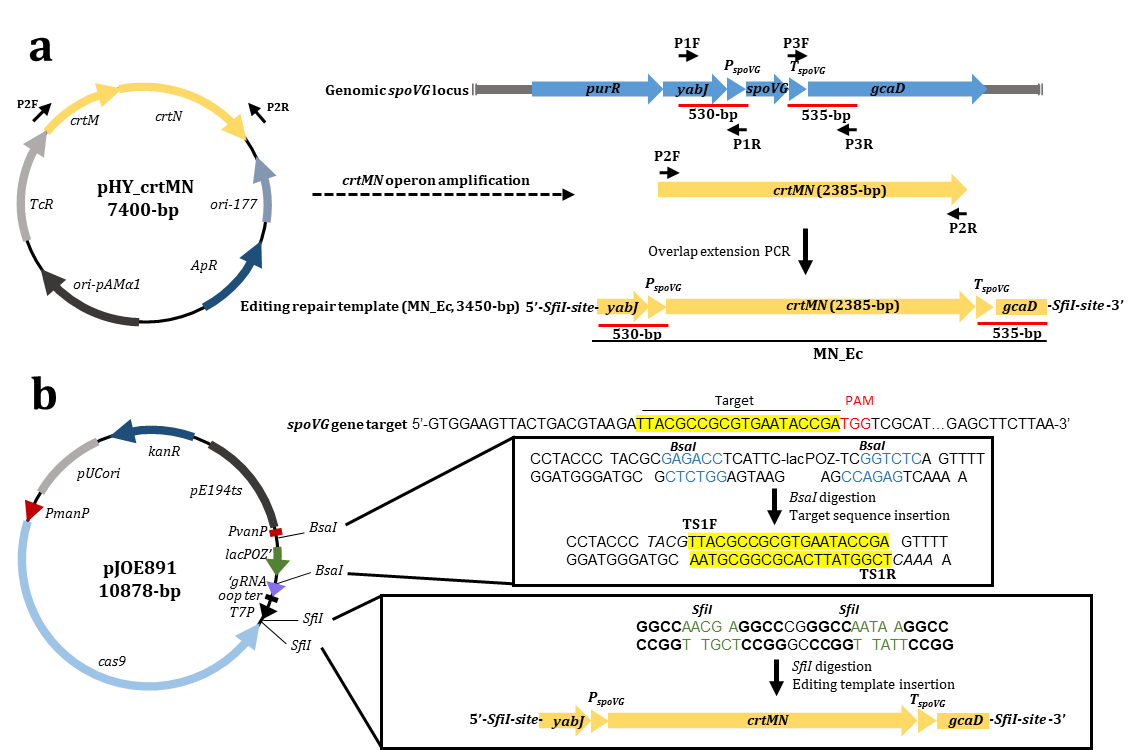


**Figure S2.** Construction system of vector pJOE891 (**a**) Diagram showing the editing repair template construction (MN_Ec) design consisting of *crtMN* operon flanked by *spoVG* homologous arms. (**b**) Physical map of pJOE891 vector containing pUCori, *E. coli* replication origin; pE194ts, *B. subtilis* temperature-sensitive replication origin; *kanR*, kanamycin resistance marker; *cas9*, Cas9 encoding gene; P*_manP_*, mannose-inducible promoter of Cas9 encoding gene; ‘gRNA, binding scaffold for Cas9; P*_vanP_*, semisynthetic promoter of the sgRNA; *lacPOZ’*, *lacZ* α fragment; oop ter, λ *oop* terminator; T7P, T7 promoter. Insets show the insertion of *spoVG* gene target sequence (TS1F/TS1R primers) and editing repair template (3450-bp) in the *BsaI* and *SfiI* sites of the pJOE891 vector, respectively.

**
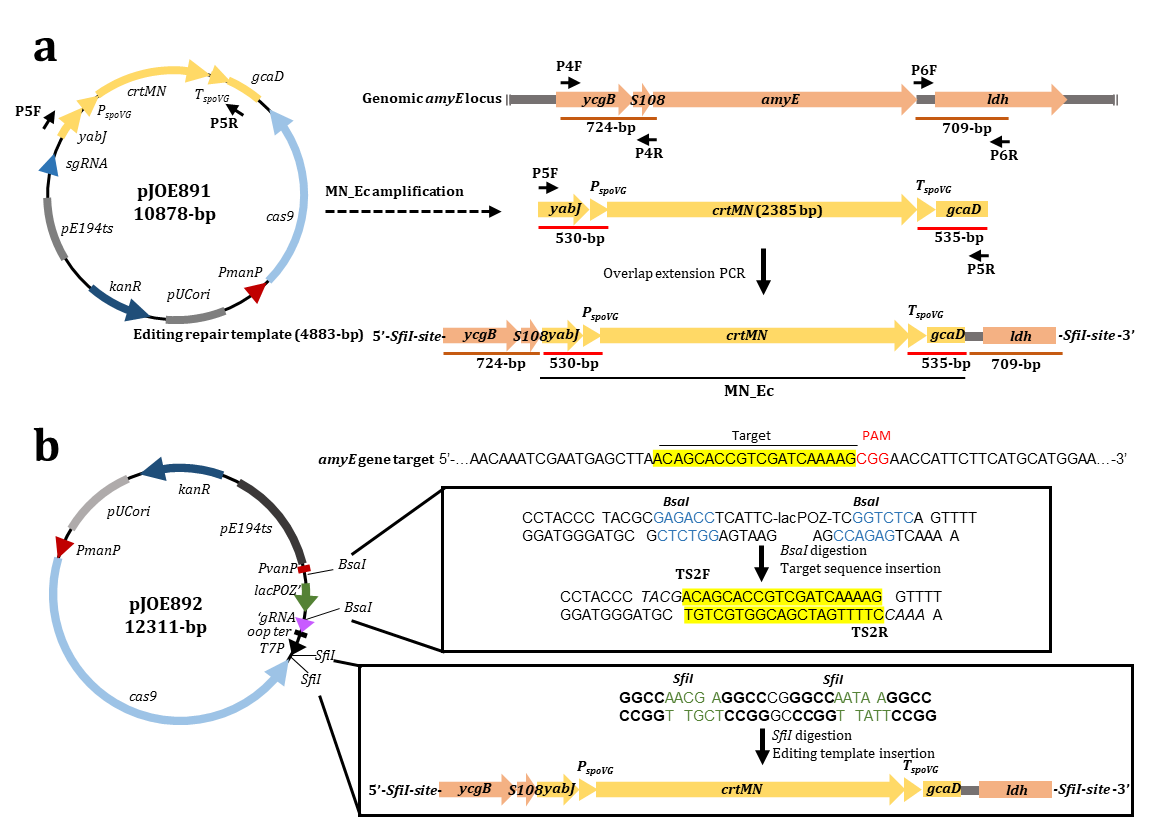
**

**Figure S3**. Construction system of vector pJOE892. (**a**) Diagram showing the editing repair template construction design consisting of MN_Ec flanked by *amyE* homologous arms. (**b**) Physical map of pJOE892 vector. Insets show the insertion of *amyE* gene target sequence (TS2F/TS2R primers) and editing repair template (4883-bp) in the *BsaI* and *SfiI* sites of the pJOE892 vector, respectively.

**
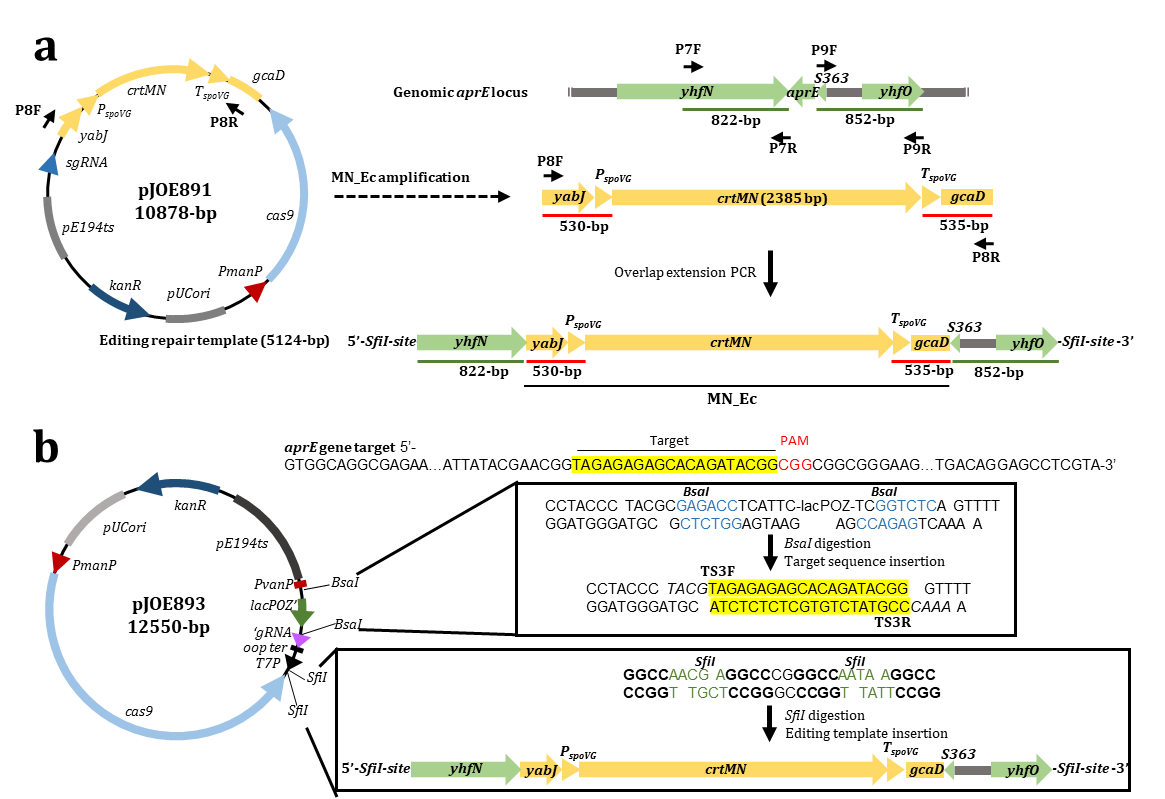
**

**Figure S4**. Construction system of vector pJOE893. (**a**) Diagram showing the editing repair template construction consisting of MN_Ec flanked by *aprE* homologous arms. (**b**) Physical map of pJOE893 vector. Insets show the insertion of *aprE* gene target sequence (TS3F/TS3R primers) and editing repair template (5124-bp) in the *BsaI* and *SfiI* sites of the pJOE893 vector, respectively.

**
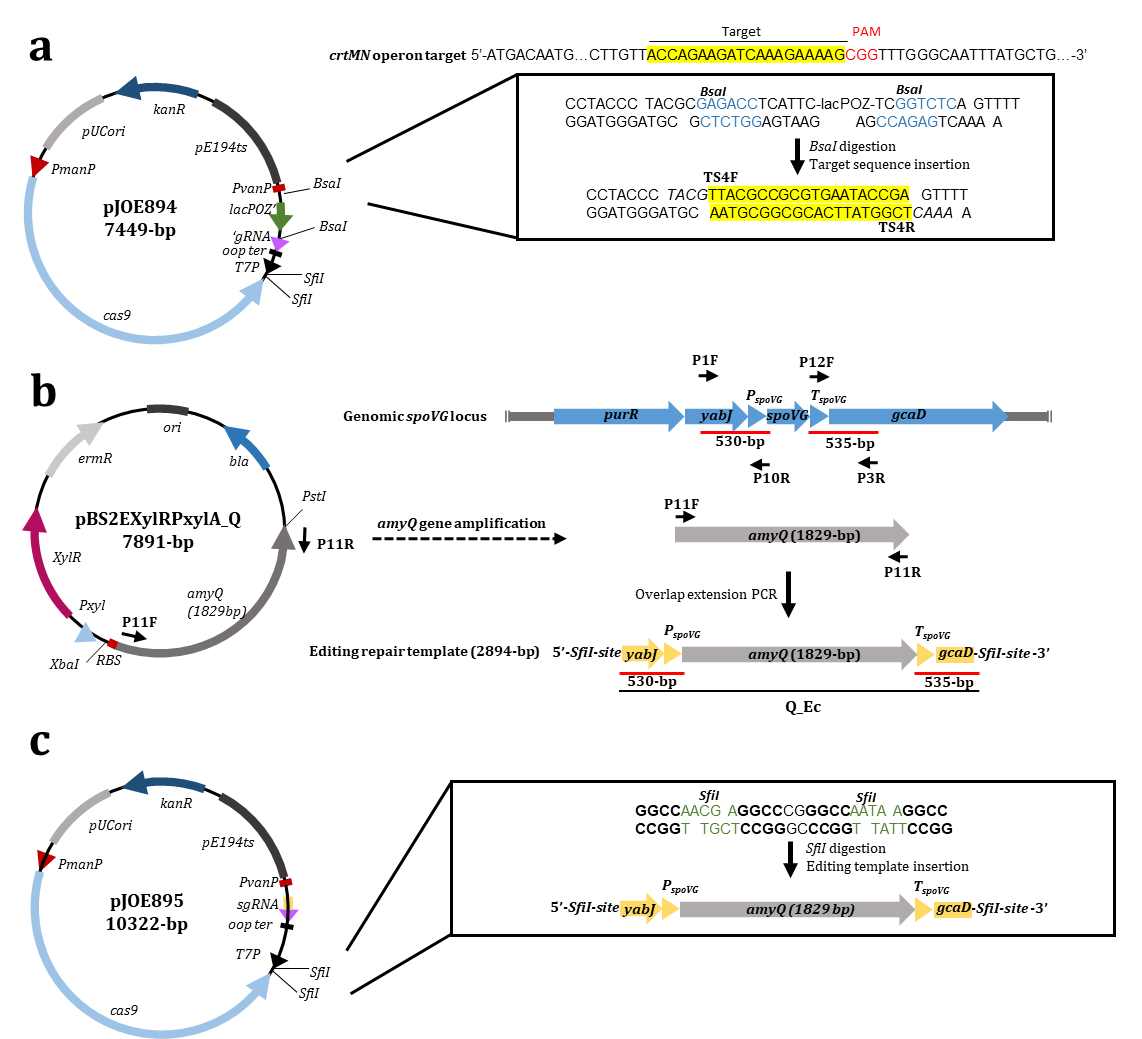
**

**Figure S5**. Construction system of vector pJOE894. (**a**) Construction system of pJOE894 plasmid. Inset show the insertion of *crtMN* operon target sequence (TS4F/TS4R primers) in the *BsaI* site of the pJOE8999 vector. (**b**) Diagram showing the editing repair template (Q_Ec) construction design consisting of *amyQ* gene (1829-bp) flanked by *spoVG* homologous arms (**c**) Physical map of pJOE895. Inset show the insertion of Q_Ec in the *SfiI* site of the pJOE895 vector.

**
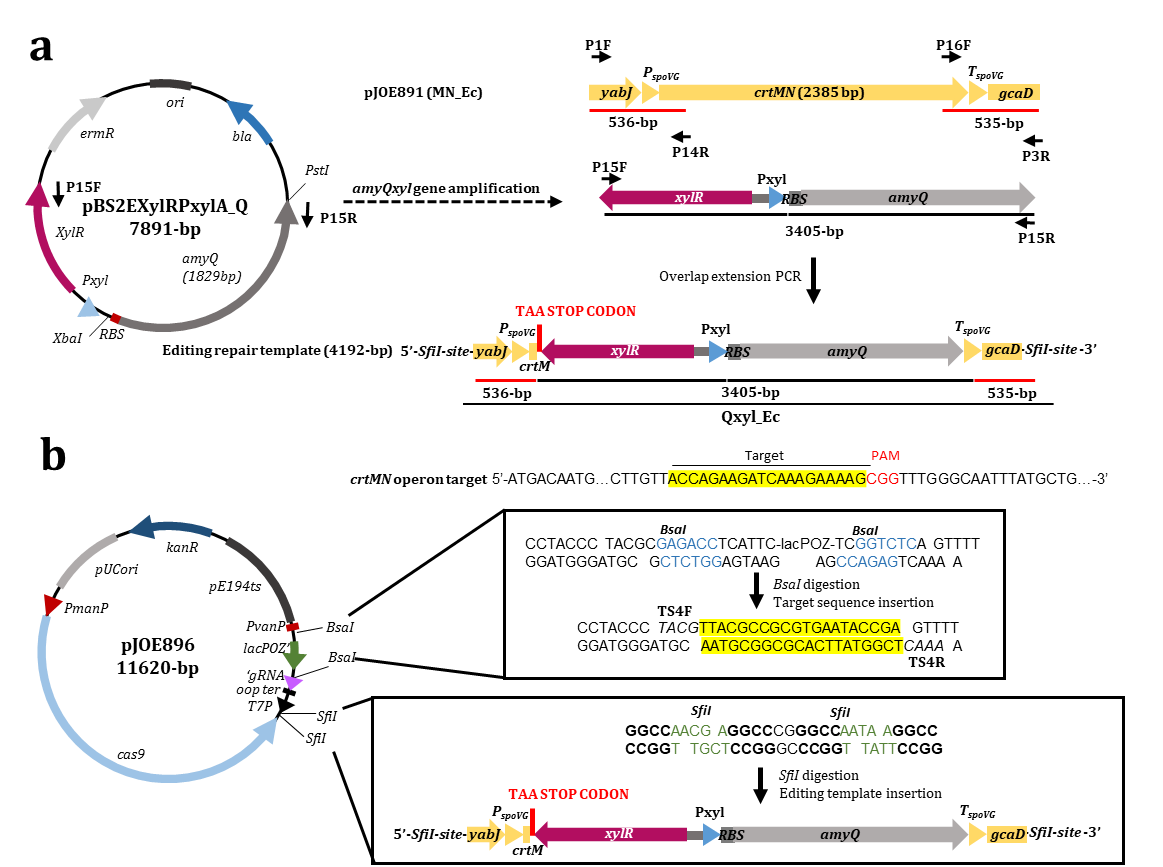
**

**Figure S6**. Construction system of vector pJOE896. (**a**) Diagram showing the editing repair template construction (Qxyl_Ec) design consisting of *amyQxyl* gene (3405-bp) flanked by *spoVG* homologous arms. (**b**) Physical map of pJOE896 vector. Insets show the insertion of *crtMN* operon target sequence (TS4F/TS4R primers) and Qxyl_Ec in the *BsaI* and *SfiI* sites of the pJOE896, respectively.

**Figure S7.** Standard calibration curve for the determination of maltose released in the α-amylase assay.
