# Supplementary material for: Barriers to simultaneous multilocus integration in Bacillus subtilis tumble down: development of a straightforward screening method for the colorimetric detection of one-step multiple gene insertion using the CRISPR-Cas9 system
Source: Microb Cell Fact. 2023 Jan 31;22:21. doi: 10.1186/s12934-023-02032-2 (PMC9890709; doi:10.1186/s12934-023-02032-2)
Supplement: Supplementary file 2 — Additional file 2: Table S1. Plasmids used in this study. Table S2. Primers designed in this study. Table S3. Splicing with overlap extension PCR (SOEing-PCR) program. [file 12934_2023_2032_MOESM2_ESM.docx]

**Barriers to simultaneous multilocus integration in *Bacillus subtilis* tumble down: development of a straightforward screening method for the colorimetric detection of one-step multiple gene insertion using the CRISPR-Cas9 system**

Jordi Ferrando, Oriana Filluelo, Daniel R Zeigler and Pere Picart

**SUPPLEMENTARY MATERIAL.**

**Additional file 2**

**Table S1**: Plasmids used in this study

**Table S2**: Primers designed in this study

**Table S3**: Splicing with overlap extension PCR (SOEing-PCR) program

**Table S1.** Plasmids used in this study.

| **Plasmid** | **Description** | **Source/Reference** |
| --- | --- | --- |
| pHY_crtMN | Plasmid pHY300PLK containing the *crtMN* operon from *Staphylococcus aureus* | (1) |
| pJOE8999 | P*_manP-cas9_*, pUC, pE194^ts^, *kan*^r^ | (2) |
| pBS2EXxylRPxylA | Plasmid containing the xylose-inducing promoter xylose-repressor system | (3) |
| pBS2EXxylRPxylA_Q | Plasmid pBS2EXxylRPxylA containing the *amyQ* gene | This study |
| pJOE891 | Plasmid used to replace the *spoVG* locus for MN_Ec | This study |
| pJOE892 | Plasmid used to replace the *amyE* locus for MN_Ec | This study |
| pJOE893 | Plasmid used to replace the *aprE* locus for MN_Ec | This study |
| pJOE894 | Plasmid used to confirm that the NHEJ system is not active in *B. subtilis* | This study |
| pJOE895 | Plasmid used for the simultaneous insertion of multiple Q_Ec copies | This study |
| pJOE896 | Plasmid used for the simultaneous insertion of multiple Qxyl_Ec copies | This study |
|  |  |  |

**Table S2**. Primers designed in this study.

| **Name** | **Sequence** | **Purpose** | **PCR Product** | |
| --- | --- | --- | --- | --- |
| **TS1F** | 5' – TACGTTACGCCGCGTGAATACCGA | **Target sequence for *spoVG* gene replacement** |  |  |
| **TS1R** | 5' – AAACTCGGTATTCACGCGGCGTAA |  |  | |
|  |  | **PCR of homology template for *spoVG* replacement for *crtMN* operon (MN_Ec)** | **3450-bp** | |
| **P1F** | 5’ – AAGGCCAACGAGGCCCGGGCCTTATTCACAAGGG | upstream *spoVG* gene homologous arm | 530-bp | |
| **P1R** | 5’ – AAATTCATATCCATCATTGTCATAGTAGTTCACCACCTTTTC |  |  |  |
| **P2F** | 5’ – GAAAAGGTGGTGAACTACTATGACAATGATGGATATGAATTT | *crtMN* operon amplification | 2385-bp | |
| **P2R** | 5’ – TCCTTGCTTTTTGGTTATTTTTTATACGCCCCGCTCAAT |  |  |  |
| **P3F** | 5’ – ATTGAGCGGGGCGTATAAAAAAATAACCAAAAAGCAAGGA | downstream *spoVG* gene homologous arm | 535-bp | |
| **P3R** | 5’ – AAGGCCTTATTGGCCTTACTGTTCCATCGTCTCTGCTG |  |  |  |
| **TS2F** | 5’ – TACGACAGCACCGTCGATCAAAAG | **Target sequence for amyE gene replacement** |  | |
| **TS2R** | 5’ – AAACCTTTTGATCGACGGTGCTGT |  |  | |
|  |  | **PCR of homology template for *amyE* replacement for *crtMN* operon** | **4883-bp** | |
| **P4F** | 5’ – AAGGCCAACGAGGCCGAGTTATTCATTGCAGAAGCGCA | upstream *amyE* gene homologous arm | 724-bp | |
| **P4R** | 5’ – ACCGATGTGAAGACTGGAGAA |  |  |  |
| **P5F** | 5’ – TTCTCCAGTCTTCACATCGGTTCGGGCCTTATTCACAAGGG | Expression cassete containing *crtMN* operon amplification (MN_Ec) | 3450-bp | |
| **P5R** | 5’ – TTACTGTTCCATCGTCTCTGCTG |  |  |  |
| **P6F** | 5’ – CAGCAGAGACGATGGAACAGTAAGCGGTTCTCTTCCCCATTGA | downstream *amyE* gene homologous arm | 709-bp | |
| **P6R** | 5’ – AAGGCCTTATTGGCCTACGTTTTGAGGCGCTGCG |  |  |  |
| **TS3F** | 5’ – TACGTAGAGAGAGCACAGATACGG | **Target sequence for *aprE* gene replacement** |  | |
| **TS3R** | 5’ – AAACCCGTATCTGTGCTCTCTCTA |  |  | |
|  |  | **PCR of homology template for *aprE* replacement for *crtMN* operon** | **5124-bp** | |
| **P7F** | 5’ – AAGGCCAACGAGGCCGCTCATACAAGCTTCTTGCC | upstream *aprE* gene homologous arm | 822-bp | |
| **P7R** | 5’ – CACTCTTTACCCTCTCCTTTTAA |  |  |  |
| **P8F** | 5’ – TTAAAAGGAGAGGGTAAAGAGTGTCGGGCCTTATTCACAAGGG | Expression cassete containing *crtMN* operon amplification (MN_Ec) | 3450-bp | |
| **P8R** | 5’ – TTACTGTTCCATCGTCTCTGCTG |  |  |  |
| **P9F** | 5’ – CAGCAGAGACGATGGAACAGTAATAGTAAAAAGAAGCAGGTTCCT | downstream *aprE* gene homologous arm | 852-bp | |
| **P9R** | 5’ – AAGGCCTTATTGGCCCTTTACGCTTTGCGTTCTCG |  |  |  |
|  |  |  |  | |

**Table S2**. Primers designed in this study (continued).

| **Name** | **Sequence** | **Purpose** | **PCR Product** |
| --- | --- | --- | --- |
| **TS4F** | 5' – TACGACCAGAAGATCAAAGAAAAG | **Target sequence for *crtMN* operon replacement** |  |
| **TS4R** | 5’ – AAACCTTTTCTTTGATCTTCTGGT |  |  |
|  |  | **PCR of homology template for crtMN *operon* replacement for amyQ *gene* (Q_Ec)** | **2894-bp** |
| **P1F** | 5’ – AAGGCCAACGAGGCCCGGGCCTTATTCACAAGGG | upstream *spoVG* gene homologous arm | 530-bp |
| **P10R** | 5’ – CTTTCGTTTTTGAATCATAGTAGTTCACCACCTTTTCCC |  |  |
| **P11F** | 5’ – GTGAACTACTATGATTCAAAAACGAAAGCGGAC | *amyQ* gene amplification | 1829-bp |
| **P11R** | 5’ – GGTTATTTTCACGTTGTGATTAAAAGCAGCG |  |  |
| **P12F** | 5’ – GCTTTTAATCACAACGTGAAAATAACCAAAAAGCAAGGACTG | downstream *spoVG* gene homologous arm | 535-bp |
| **P3R** | 5’ – AAGGCCTTATTGGCCTTACTGTTCCATCGTCTCTGCTG |  |  |
|  |  | **Cloning *the* amyQ gene in plasmid pBS2EXxylRPxylA *using* XbaI-PstI sites** |  |
| **P13F** | 5' – GGTCTAGAGATAAGAAAGGGAGGACAAACA | *amyQ* gene with ribosome-binding site amplification | 1850-bp |
| **P13R** | 5' – TACTGCAGCACGTTGTGATTAAAAGCAGCG |  |  |
|  |  | **PCR of homology template for crtMN *operon* replacement for amyQ-xylR gene (Qxyl_Ec)** | **4670-bp** |
| **P1F** | 5' – AAGGCCAACGAGGCCCGGGCCTTATTCACAAGGG | upstream *spoVG* site homologous arm | 630-bp |
| **P14R** | 5' – TTATTGATCTTCTGGTAACAAGTCA |  |  |
| **P15F** | 5' – TGACTTGTTACCAGAAGATCAATAATGGCCGACTTTAGATATTTTCGT | *amyQxyl* gene amplification | 3405-bp |
| **P15R** | 5' – TGGATGCGTACTTGCACCTGCCACGTTGTGATTAAAAGCAGCG |  |  |
| **P16F** | 5' – GCAGGTGCAAGTACGCATCCA | downstream *spoVG* site homologous arm | 635-bp |
| **P3R** | 5’ – AAGGCCTTATTGGCCTTACTGTTCCATCGTCTCTGCTG |  |  |
|  |  | **Validation primers used in the genome editing experiments** |  |
| **P17F** | 5' – TTCAGGCTCCTCAAACCGCA | PCR verification with outside primers for gene insertion at *spoVG* site | 4134-bp (*crtMN* operon) |
| **P17R** | 5' – TTACCAGTCTGGTAAGCGGC |  | 3578-bp (*amyQ* gene) |
|  |  |  | 5154-bp (*amyQxyl* gene) |
| **P18F** | 5' – GAGTTATTCATTGCAGAAGCGCA | PCR verification with outside primers for gene insertion at *amyE* site | 4883-bp (*crtMN* operon) |
| **P18R** | 5' – TACGTTTTGAGGCGCTGCG |  | 4327-bp (*amyQ* gene) |
|  |  |  | 6098-bp (*amyQxyl* gene) |
| **P19F** | 5' – GCTCATACAAGCTTCTTGCC | PCR verification with outside primers for gene insertion at *aprE* site | 5124-bp (*crtMN* operon) |
| **P19R** | 5' – CTTTACGCTTTGCGTTCTCG |  | 4568-bp (*amyQ* gene) |
|  |  |  | 6339-bp (*amyQxyl* gene) |
|  |  |  |  |

**Table S3**. Splicing with overlap extension PCR (SOEing-PCR) program.

| **OVERLAP EXTENSION PCR** | **TEMPERATURE** | **TIME** |
| --- | --- | --- |
| **STEP 1** |  |  |
| Initial Denaturation | 95°C | 5 minutes |
|  | 95°C | 30 seconds |
| 15 cycles | X °C * | 1:30 minutes |
|  | 72°C | X minutes ** |
| Final Extension | 72°C | 5 minutes |
| **STEP 2** |  |  |
| Initial Denaturation | 95°C | 5 minutes |
|  | 95°C | 30 seconds |
| 30 cycles | X °C * | 30 seconds |
|  | 72°C | X minutes ** |
| Final Extension | 72°C | 5 minutes |
| Hold | 4 °C |  |
| ** Depending on the melting temperature (Tm) of the primer | | |
| ** Depending on the length of the fragment to be amplified | | |

**References**

1. Yoshida K, Ueda S, Maeda I. Carotenoid production in *Bacillus subtilis* achieved by metabolic engineering. Biotechnol Lett. 1, 1789-1793 (2009).
2. Altenbuchner J. Editing of the *Bacillus subtilis* Genome by the CRISPRCas9 System. Appl Environ Microbiol. 82, 5421–5427 (2016).
3. Popp PF, Dotzler M, Radeck J, Bartels J, Mascher T. The *Bacillus* BioBrick Box 2.0: expanding the genetic toolbox for the standardized work with *Bacillus subtilis*. Sci Rep. 7, 15058-15070 (2017).
